# Hepatic ACSL4 Loss Boosts Endogenous Gamma-Glutamylcysteine to Alleviate Alcoholic Liver Disease

**DOI:** 10.3390/antiox15040438

**Published:** 2026-03-31

**Authors:** Ran Duan, Xin-Yi Wang, Xue Zhou, Jing-Wen Ding, Zhi-Sen Yang, Zhi-Lin Li, Yue-Yu Wang, Jia-Xin Yu, Jing-Jing Duan

**Affiliations:** Institute of Translational Medicine, School of Basic Medicine and Clinical Pharmacy, China Pharmaceutical University, Nanjing 211198, China; 3121090273@stu.cpu.edu.cn (R.D.); 18052868875@163.com (X.-Y.W.); zhouxue202103@163.com (X.Z.); dingjw0206@163.com (J.-W.D.); 3323092138@stu.cpu.edu.cn (Z.-S.Y.); sdrzlslzl@163.com (Z.-L.L.); wyycpu1272@163.com (Y.-Y.W.); 18852617619@163.com (J.-X.Y.)

**Keywords:** alcoholic liver disease, lipid metabolism, lipid peroxidation, small molecule bioactive peptide-mediated antioxidant system, MAPK signaling pathway

## Abstract

Alcoholic liver disease (ALD), secondary to chronic alcohol abuse, encompasses a spectrum of liver disorders that progress from steatosis and hepatitis to fibrosis, cirrhosis, and acute-on-chronic liver failure. It poses a considerable global health burden due to its elevated rates of associated morbidity and mortality. The rising prevalence of ALD, coupled with the lack of approved pharmacotherapies, presents considerable unmet clinical needs. In this study, long-chain acyl-CoA synthetase 4 (ACSL4) was identified as a pathogenic driver in the context of chronic alcohol consumption. Hepatocyte *Acsl4* ablation mitigated key pathological manifestations in Gao-Binge model mice, as evidenced by reduced inflammatory cell infiltration and attenuated lipid accumulation. Mechanistically, ACSL4 inhibition augmented cellular antioxidant defence through elevating gamma-glutamylcysteine (γ-GC) levels. In addition, γ-GC bound to and suppressed the expression of protein tyrosine phosphatase type IVA member 1 (PTP4A1). Both genetic silencing and pharmacological inhibition of PTP4A1 attenuated the activation of the downstream MAPK-NF-κB inflammatory cascade. Dronedarone, identified as a novel compound targeting ACSL4, demonstrated efficacy in ameliorating the progression of ALD. Overall, these findings elucidate a novel mechanism wherein ACSL4 modulates antioxidant responses via a small bioactive peptide, highlighting ACSL4 as a potential therapeutic target for ALD.

## 1. Introduction

Alcoholic liver disease (ALD) represents a major cause of chronic liver disease worldwide [1]. In recent years, ALD has emerged as a global health concern that imposes a significant economic burden and public health threat, attributable to its rising incidence and mortality rates [2]. Specifically, ALD encompasses a broad spectrum of disorders, ranging from simple steatosis to severe liver injury, including alcoholic hepatitis (AH), cirrhosis, and hepatocellular carcinoma (HCC) [1]. To date, effective biomarkers and clinical treatment options for ALD remain scarce. Thus, abstinence from alcohol remains the most efficient approach for the management of ALD [3]. Available pharmacotherapies, such as S-adenosyl-L-methionine [4], corticosteroids [5], and pentoxifylline [6], may partially relieve ALD symptoms. However, given their narrow therapeutic windows and suboptimal safety profiles, the identification and development of novel therapeutic targets represent an urgent priority in ALD management.

Complicated risk factors have been implicated in the progression of ALD, especially dysregulated lipid metabolism and oxidative stress. Alcohol exposure disrupts hepatic lipid homeostasis by simultaneously promoting lipogenesis and impairing fatty acid oxidation [7,8,9,10]. Disruption of fatty acid metabolism induces an overproduction of reactive oxygen species (ROS) that surpasses the cellular antioxidant capacity, resulting in exacerbated oxidative stress. Under these conditions, ROS attack polyunsaturated fatty acids (PUFAs), leading to the generation of toxic lipid peroxides (LPOs) that trigger cellular damage and death. As a central regulator of fatty acid metabolism, long-chain acyl-CoA synthetase 4 (ACSL4) catalyzes the incorporation of PUFAs—such as arachidonic acid (AA)—into cell membranes [11]. Elevated ACSL4 expression increases the susceptibility of cellular membranes to lipid peroxidation, underscoring its role as a critical mediator and indicator of ferroptosis [12,13]. Previous studies have unraveled the role and molecular mechanism of ACSL4 in mediating fatty acid metabolism and lipid peroxidation in metabolic dysfunction-associated steatotic liver disease (MASLD) [14]. Moreover, emerging evidence has revealed the involvement of ACSL4 in various forms of tissue injury, including brain injury, neuroinflammation [15,16], intestinal ischemia–reperfusion injury [17], and acute kidney injury [18]. These findings establish ACSL4 as a key driver in the pathogenesis of injury-related liver diseases.

Given the therapeutic gap in ALD management, we sought to identify key pathogenic drivers and therapeutic targets. Through clinical database analysis, we observed that ACSL4 expression is significantly upregulated in the livers of patients with alcoholic liver injury and correlates with poor prognosis in alcohol-exposed HCC patients, prompting us to investigate its functional role in ALD progression. Using hepatocyte-specific *Acsl4* knockout mice (*Acsl4*^HKO^), we found that ACSL4 deficiency protects against alcohol-induced steatosis, lipid peroxidation, and inflammatory infiltration. Mechanistically, we identified a previously unrecognized ACSL4/γ-GC/PTP4A1 axis, wherein ACSL4 inhibition elevates gamma-glutamylcysteine (γ-GC) levels, which in turn suppresses protein tyrosine phosphatase type IVA member 1 (PTP4A1) activity and dampens downstream MAPK-NF-κB inflammatory signaling. Moreover, through computational screening, we discovered dronedarone—an FDA-approved antiarrhythmic drug—as a novel ACSL4-targeting compound capable of mitigating ALD pathology in vivo. Collectively, these findings provide new insights into the mechanism by which ACSL4 regulates ferroptosis through a bioactive peptide antioxidant system and unveil potential therapeutic strategies for ALD.

## 2. Materials and Methods

### 2.1. Mouse Experiments

C57BL/6j mice were obtained from Gempharmatech Co., Ltd. (Nanjing, China). Albumin–Cre mice and Floxed mice were established at the Shanghai Model Organisms Center, Inc. (Shanghai, China). *Acsl4*^flox^ mice were crossed with Albumin–Cre transgenic mice through a series of steps to generate hepatocyte-specific *Acsl4* knockout (*Acsl4*^HKO^) mice. The primers used for gene identification are shown in Supplementary Table S2.

For the establishment of the Gao-Binge model, all mice (male, 7–8 weeks of age) were fed the Lieber–DeCarli pair-fed diet (TP4030D, TrophicDiet, Nantong, China) for 5 days to become acclimated to a liquid diet [19]. EtOH-fed group was then fed with a Lieber–DeCarli diet containing increasing concentrations of ethanol, up to 5% (*v*/*v*), provided ad libitum for 20 days. Control diet (TP4030C, TrophicDiet) was supplemented with an isocaloric amount of maltose (pair-fed). Pair-fed mice were calorie-matched with the ethanol-fed mice. After 20 days of feeding, the EtOH-fed group was gavaged with alcohol (volume fraction of 31.5%, mass concentration of 5 g/kg), and the isocaloric liquid diet control group was gavaged with maltodextrin (volume fraction of 45%, mass concentration of 9 g/kg). Eight hours after gavage the mice were euthanised. Blood, tissue samples of liver and intestine were collected afterwards.

For the Gao-Binge model mice administered with JMS-053, the Lieber–DeCarli diet was provided for 10 days, with intraperitoneal injection of 5 mg/kg or 10 mg/kg JMS-053 once daily starting from day 6. For the Gao-Binge model mice administered with dronedarone, the Lieber–DeCarli diet was provided for 10 days, with oral gavage of 50 mg/kg or 100 mg/kg dronedarone once daily starting from day 6. Doses were determined based on preliminary experiments.

### 2.2. Cell Culture

To establish cells with stable knockdown of ACSL4, HepG2 and AML12 cells were infected with lentiviruses carrying shRNAs targeting human or mouse *Acsl4*. The target sequences were as follows: for human ACSL4, GCAGTAGTTCATGGGCTAAAT (LV2N-*ACSL4*-homo-1) and GCAGAGATATCTTGCTTTACC (LV2N-*ACSL4*-homo-2); for mouse *Acsl4*, GCAGAAGATTATTGTGTTGAT (Mus *Acsl4*-shRNA-1) and GCAAGGTTCAAGAGATGAATT (Mus *Acsl4*-shRNA-2). A non-targeting shRNA (shNC; LV2N-NC-homo and Mus NC shRNA: TTCTCCGAACGTGTCACGT) was used as a control. Subsequently, cells were selected with 3 μg/mL puromycin for two weeks. The knockdown lentivirus was constructed by Suzhou GenePharma Co., Ltd. (Suzhou, China).

### 2.3. Metabolic Studies in Mice

In Gao-Binge model *Acsl4*^flox^ and *Acsl4*^HKO^ mice, levels of alanine aminotransferase (ALT) and aspartate aminotransferase (AST) were assessed using the Alanine Aminotransferase Assay Kit and the Aspartate Aminotransferase Assay Kit (C009-2-1, C010-2-1, Jiancheng, Nanjing, China), respectively. The contents of triglycerides (TG) and total cholesterol (TCHO) in serum and liver were measured using the Triglyceride Assay Kit (A110-1-1, Jiancheng) and the Total Cholesterol Assay Kit (A111-1-1, Jiancheng). The liver TG and TCHO contents were normalized to the liver weight. All procedures were carried out according to the manufacturer’s instructions.

In JMS-053-treated or Dron-treated Gao-Binge model mice, whole blood and serum samples were collected for complete blood count (CBC) and blood biochemistry tests, respectively. The results were analyzed using a Siemens Advia Xpand Plus biochemical analyzer (Siemens; Munich, Germany).

The levels of Malondialdehyde (MDA), superoxide dismutase (SOD), catalase (CAT), and glutathione (GSH) in mouse liver tissue were quantified using commercial assay kits and normalized to liver weight. Measurements were performed following the manufacturer’s protocols with the following kits: Malondialdehyde Assay Kit (TBA method, A003-1-2; Jiancheng), Superoxide Dismutase Assay Kit (WST-1 method, A001-3-1; Jiancheng), Catalase Assay Kit (A007-1-1; Jiancheng), and Reduced Glutathione Assay Kit (A006-2-1; Jiancheng).

### 2.4. Histopathological Analysis

For hematoxylin and eosin (H&E) staining, after formalin fixation and automated dehydration, paraffin-embedded liver sections from mice (4 μm) were dewaxed and rehydrated through sequential xylene and graded ethanol solutions. The sections were stained with filtered hematoxylin, differentiated in 1% acid alcohol, and blued in 0.5% ammonia solution. After eosin staining, the tissues were dehydrated through a graded ethanol series and xylene, then mounted with neutral balsam.

The expression levels of ACSL4 (1:100; ab155282, Abcam, Cambridge, UK), and myeloperoxidase (MPO; 1:200; 22225-1-AP, Proteintech, Rosemont, IL, USA) were determined by immunohistochemistry (IHC) staining.

Frozen liver sections (8 μm) were stained with Oil Red O solution (O9755, Sigma, St. Louis, MO, USA), followed by differentiation in isopropanol, hematoxylin counterstaining, and mounting with glycerin gelatin.

Whole-slide scanned images (WSIs) have been captured to address potential concerns about regional heterogeneity. The original TIFF-format WSIs can be provided upon request.

### 2.5. Primary Mouse Hepatocyte Isolation

Primary mouse hepatocytes were cultured in DMEM-F12 (KGM12500-500, KeyGEN BioTECH, Nanjing, China) medium containing ITS-G (1:100; 41400045, Thermo Fisher, Waltham, MA, USA) and dexamethasone (5 µg/mL; ID0170, Solarbio, Beijing, China). Initially, ethylene glycol tetraacetic acid (EGTA, E0396, Sigma) was used to chelate intracellular calcium in the liver. Subsequently, the cells were digested with collagenase (0.05%; C0130, Sigma). The digested tissue was filtered through a 70-micron cell strainer and centrifuged five times at 70 g for 3 min. The precipitate after centrifugation was resuspended and seeded onto culture dishes for use in experiments the following day.

### 2.6. Cell Treatment

Cells were seeded in 96-well or 6-well plates and treated with alcohol for 24 h for subsequent assays. Cell viability was evaluated using the cell counting kit-8 (CCK-8) kit (C0043, Beyotime, Shanghai, China), while LDH release in the supernatant was measured with the lactate dehydrogenase (LDH) Cytotoxicity Assay Kit (C0016, Beyotime), using untreated cells as the negative control. Intracellular ROS levels were detected by flow cytometry with the Reactive Oxygen Species Assay Kit (S0033S, Beyotime), with RosUp as a positive control. Lipid peroxidation was assessed using the BODIPY 581/591 C11 probe (D3861, Thermo Fisher) via flow cytometry and confocal microscopy. Mitochondrial superoxide was assessed using the MitoSOX Red probe (HY-D1055, MCE, Monmouth Junction, NJ, USA). Cellular GSH and MDA contents were determined using commercial assay kits (A006-2-1 and A003-4-1, Jiancheng), respectively, following the manufacturers’ protocols.

### 2.7. Flow Cytometry

Cell suspensions were prepared from livers of Gao-Binge model mice by digestion with Collagenase D (0.25 mg/mL; Roche, Basel, Switzerland) and DNase I (0.1 mg/mL; Sigma). After blocking with FcR Blocking Reagent (Miltenyi Biotec, Bergisch Gladbach, Germany) and lysing red blood cells, the cells were stained with surface marker antibodies on ice for 30 min. Samples were analyzed on an Attune NxT flow cytometer (Thermo Fisher), and data processing was performed using FlowJo v.10 (RRID:SCR_008520). The antibodies used are shown in Supplementary Table S4.

### 2.8. Bulk RNA-Sequencing Analysis

After the Gao-Binge model was established, *Acsl4*^HKO^ (*n* = 5) and *Acsl4*^flox^ (*n* = 5) mice were dissected, and their livers were extracted. The transcriptome sequencing was conducted by LC-Bio Technology Co., Ltd. (Hangzhou, China), with detailed methods provided in the Supplementary Materials and Methods.

### 2.9. Approved Compound Library Screening

A diverse library of 2513 approved drugs from DrugBank (Edmonton, AB, Canada) was prepared in PDBQT format using OpenBabel (3.1.1) for molecular docking. The ACSL4 protein structure was modeled with I-TASSER using RCSB templates, yielding a model with RMSD < 3 Å, and the FAD binding site was predicted to generate a virtual inhibitor profile. Molecular docking was performed with AutoDock Vina using a global docking approach across the entire protein surface. The resulting poses were evaluated using a custom “AI Triple Integral Collaborative Filtering Algorithm” that prioritized compounds based on spatial overlap with the virtual inhibitor’s binding site and superior binding free energy. Candidates meeting both criteria were selected for experimental testing.

### 2.10. Molecular Docking

For validation of candidate compounds, molecular docking was performed using AutoDock Vina (v.1.1.2; Scripps Research, La Jolla, CA, USA). The ACSL4 protein structure, obtained from AlphaFold (3.0.1), was preprocessed by removing non-protein components and adding polar hydrogens. Default parameters were applied during the docking process, and flexible docking was performed to accommodate ligand flexibility within the movable active site of ACSL4. Resulting ligand–protein complexes were visualized using PyMol 2.5. Molecular docking of the four metabolites with PTP4A1 was performed using the same protocol.

### 2.11. Cellular Thermal Shift Assay (CETSA)

HepG2 cells were treated with 20 μM dronedarone/1 mM γ-GC or DMSO control for 3 h at 37 °C, followed by heating at graded temperatures (37–69 °C, in 4 °C increments) for 3 min. After rapid cooling on ice, soluble proteins were extracted and analyzed by Western blot using anti-ACSL4/PTP4A1 antibody.

### 2.12. Microscale Thermophoresis Assay (MST)

The recombinant ACSL4 DNA-binding domain was fluorescently labeled using the Monolith NT™ RED-NHS protein labeling system (NanoTemper Technologies, Munich, Germany). A concentration gradient of each test compound (ranging from 10 mM to 100 nM) was prepared and incubated with 20 nM fluorescently labeled ACSL4 protein at ambient temperature (25 °C). The reaction mixtures were then loaded into premium-grade hydrophilic capillaries specifically designed for MST measurements. Thermophoretic movement was recorded using a Monolith NT.115 instrument (NanoTemper Technologies) with the following parameters: 20% LED excitation intensity and medium MST power setting. Binding affinity constants (Kd values) were calculated through curve fitting analysis performed with the manufacturer’s NT Analysis software package (version 1.5.41).

### 2.13. Seahorse Analysis of Oxygen Consumption Rate (OCR)

To detect the mitochondrial respiration levels in HepG2 cells, Cell Mito Stress Test was performed according to the provided protocol (Seahorse Bioscience, Billerica, MA, USA). HepG2 cells transfected with shNC and sh*ACSL4* (8 × 10^3^ cells per well) were seeded into the wells of the Seahorse XFe96 Cell Culture Microplate (Agilent Technologies, Santa Clara, CA, USA) overnight. The cells were then changed to substrate-limited medium (Seahorse XF Base Medium-plus 0.5 mM Glucose, 1.0 mM GlutaMAX, 0.5 mM Carnitine, and 1% FBS, pH 7.4). After the subsequent injection and mixing of the compounds (assay concentrations: 1.0 μM oligomycin, 2.0 μM FCCP, and 0.5 μΜ Rotenone/antimycin A), OCR was determined in all wells. After performing protein quantification on the cells, the results were analyzed using the Seahorse Wave Pro software (2022).

### 2.14. Statistical Analysis

All data were presented as mean ± SD and were analyzed using the GraphPad Prism (v. 8.3.0; GraphPad Software, La Jolla, CA, USA). For comparisons between two groups, if the data met the assumptions of normality and homogeneity of variances, a two-tailed unpaired Student’s *t*-test was applied. Otherwise, the non-parametric Mann–Whitney U test (or Wilcoxon signed-rank test for paired data) was used. For comparisons among three or more groups involving one independent variable, one-way ANOVA was employed if the assumptions were met, followed by Tukey’s post hoc test for multiple comparisons. If the assumptions were violated, the Kruskal–Wallis test followed by Dunn’s multiple comparisons test was used. For comparisons involving two independent variables, two-way ANOVA with appropriate post hoc tests was applied. In all cases, statistical significance was shown as n.s. (not significant; *p* > 0.05), * *p* < 0.05, ** *p* < 0.01, *** *p* < 0.001. Correlations were analysed with Pearson correlation analysis. ImageJ (1.54p) was used for the quantification of histology images. The *n* values in each figure legend represented the number of the sample size or repetitions. Detailed *p*-values, *n*-values, and statistical methods were listed in the corresponding figure legends.

Additional materials and methods are supplemented in Supporting Information.

## 3. Results

### 3.1. ACSL4 Expression Is Positively Correlated with ALD Progression

**Figure 1:**
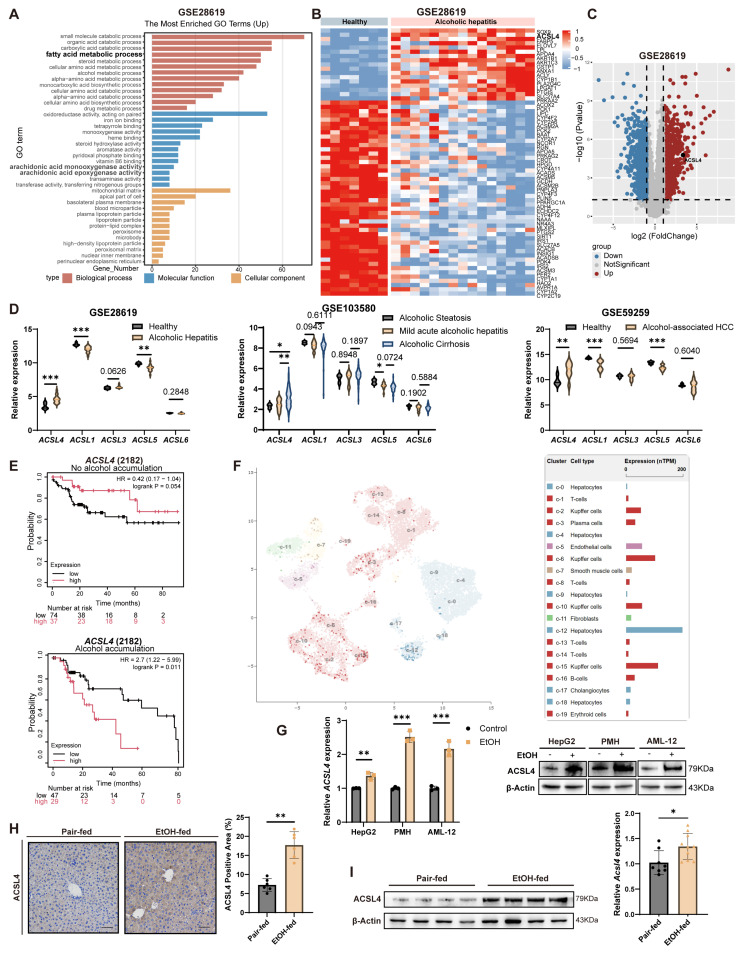
ACSL4 is upregulated in the livers of patients and mice with alcoholic liver disease. (**A**) GO enrichment analysis of DEGs in alcoholic liver disease from the GSE28619 dataset. (**B**) Heatmap of DEGs enriched in the “fatty acid metabolic process” GO term from the GSE28619 dataset. (**C**) Volcano plot of DEGs in the GSE28619 dataset. (**D**) *ACSL1*, *3*, *4*, *5*, *6* expression levels (TPM) in patients from GSE28619 (Healthy, *n* = 7; Alcoholic hepatitis, *n* = 15), GSE103580 (Alcoholic steatosis, *n* = 6, Mild acute alcoholic hepatitis, *n* = 13; Alcoholic cirrhosis, *n* = 67); and GSE59259 (Healthy, *n* = 8; Alcohol-associated HCC, *n* = 7). (**E**) Survival of HCC patients stratified by ACSL4 expression in alcohol-accumulation and non-alcohol-accumulation subgroups (TCGA-LIHC). (**F**) ACSL4 expression in human liver cell types. Source: The Human Protein Atlas. (**G**) Relative mRNA (left) and protein (right) levels of ACSL4 in HepG2, AML-12, and PMHs after EtOH exposure (100 mM) for 24 h. (**H**) Representative IHC staining (left) and quantification (right) of ACSL4 in livers from Gao-Binge model mice. Scale bar, 50 μm (*n* = 6 per group). (**I**) Relative mRNA (right) and protein (left) levels of ACSL4 in PMHs isolated from livers of Gao-Binge model mice (Pair-fed, *n* = 8; EtOH-fed, *n* = 10). Data are presented as Mean ± SD, * *p* < 0.05, ** *p* < 0.01, *** *p* < 0.001 by unpaired two-tailed Student’s *t*-test (**D**,**G**–**I**) and one-way ANOVA test (**D**).

To identify key pathways and proteins associated with ALD, we analyzed Gene Expression Omnibus (GEO: GSE28619) datasets comprising liver samples from patients with alcoholic hepatitis and healthy controls. Gene Ontology (GO) enrichment analysis of differentially expressed genes (DEGs) identified significant enrichments in the fatty acid metabolic process (Figure 1A), a finding that was further corroborated by Kyoto Encyclopedia of Genes and Genomes (KEGG) pathway analysis (Figure S1A). Among the DEGs involved in fatty acid metabolism, ACSL4 was the second most upregulated gene in AH patients (Figure 1B,C).

To further investigate the relationship between ACSL4 and alcoholic liver injury across disease stages, analysis of an additional dataset (GEO: GSE103580) revealed that ACSL4 expression progressively increased with disease severity, from alcoholic steatosis to acute alcoholic hepatitis and alcoholic cirrhosis (Figure 1D). Moreover, ACSL4 levels were significantly elevated in patients with alcohol-associated HCC (GEO: GSE59259; Figure 1D). Furthermore, other ACSL family members did not show a consistent correlation with ALD progression, suggesting that ACSL4 plays a specific role in this context (Figure 1D). Analysis of The Cancer Genome Atlas (TCGA) database unveiled that among HCC patients with a history of alcohol consumption, those exhibiting high ACSL4 expression had shorter overall survival compared to those with low expression. Conversely, in patients without alcohol consumption, high ACSL4 expression was associated with a survival benefit (Figure 1E). Additionally, single-cell RNA sequencing data of liver tissue revealed predominant expression of ACSL4 in hepatocytes, which provided the rationale for subsequent studies utilizing hepatocyte-based cellular models and hepatocyte-specific genetic editing approaches (Figure 1F).

To validate ACSL4 expression changes during alcoholic liver injury in vitro, we treated HepG2, AML-12, and primary mouse hepatocytes (PMHs) with a gradient of ethanol concentrations and assessed cytotoxicity (Figure S1B). After 24 h treatment with 100 mM ethanol or control medium (FBS-free), ACSL4 levels were significantly higher in alcohol-treated cells than in controls (Figure 1G and Figure S1C). In vivo experiments using the Gao-Binge model showed that ACSL4 expression was significantly upregulated in the PMHs from ethanol-treated mice compared to controls (Figure 1H,I and Figure S1D).

In summary, integrated bioinformatic analysis and experimental validation both in vitro and in vivo consistently demonstrate that ACSL4 expression is significantly upregulated during the progression of ALD.

### 3.2. Hepatocyte-Specific Acsl4 Knockdown Alleviates Alcohol-Induced Liver Pathology and Inflammation in Mice

**Figure 2:**
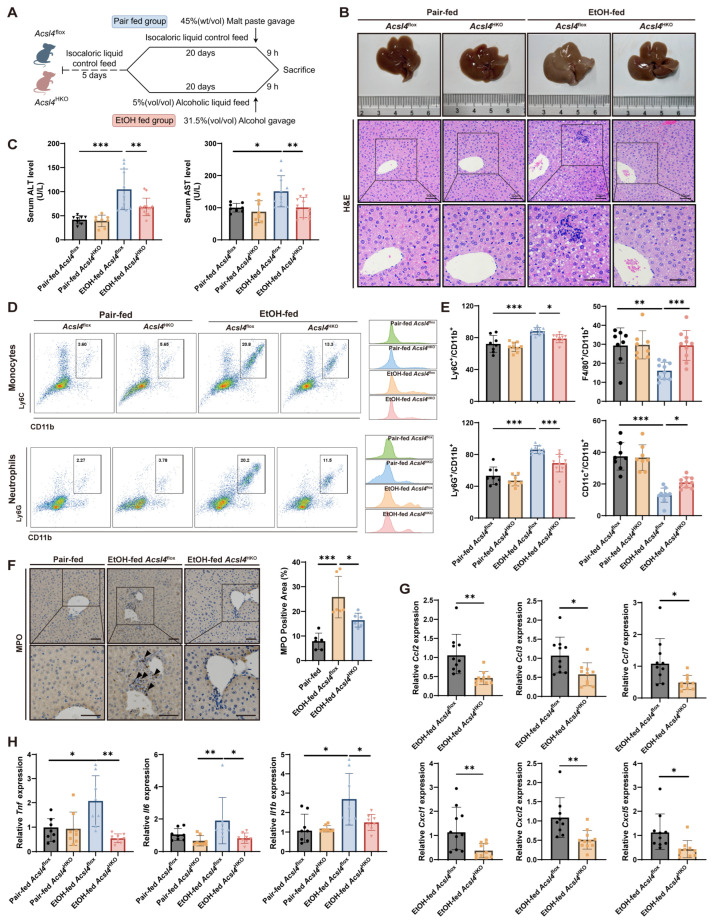
Hepatocyte-specific deletion of *Acsl4* attenuates liver injury and mitigates inflammation in mice subjected to the Gao-Binge model. (**A**–**H**): Gao-Binge model *Acsl4*^flox^ and *Acsl4*^HKO^ mice. (**A**) Gao-Binge model scheme in *Acsl4*^flox^ and *Acsl4*^HKO^ mice. (**B**) Representative images of liver morphology and H&E staining. Scale bar, 50 μm (*n* = 6 per group). (**C**) Serum ALT and AST levels (Pair-fed *Acsl4*^flox^, *n* = 8; Pair-fed *Acsl4*^HKO^, *n* = 8; EtOH-fed *Acsl4*^flox^, *n* = 11; EtOH-fed *Acsl4*^HKO^, *n* = 13). (**D**) Flow cytometric analysis of hepatic myeloid cells. Representative density plots (left) and histograms (right) showing the expression of Ly6C^+^CD11b^+^ and Ly6G^+^CD11b^+^ cells. Cells were gated on live, singlet, CD45^+^ cells. (**E**) Proportional analysis of hepatic myeloid cell populations. Frequencies of Ly6C^+^ monocytes (CD11b^+^Ly6C^+^), neutrophils (CD11b^+^Ly6G^+^), macrophages (CD11b^+^F4/80^+^), and dendritic cells (CD11b^+^CD11c^+^) among CD11b^+^ cells. All populations were gated on live, singlet, CD45^+^ immune cells. (**F**) Representative images of IHC staining of neutrophils (MPO) in livers. Scale bar, 50 μm (*n* = 6; Arrows indicate MPO-positive cells). (**G**) Relative mRNA levels of monocyte chemokines (*Ccl2*, *Ccl3*, *Ccl7*) and neutrophil chemokines (*Cxcl1*, *Cxcl2*, *Cxcl5*) in the liver (*n* = 10 per group). (**H**) Relative mRNA levels of inflammatory factors (*Tnf*, *Il1b*, *Il6*) in livers (*n* = 8). Data are presented as the mean ± SD, * *p* < 0.05, ** *p* < 0.01, *** *p* < 0.001 by unpaired two-tailed Student’s *t*-test (**G**) and one-way ANOVA test (**C**,**E**,**F**,**H**).

To investigate the role of ACSL4 in liver function during alcohol exposure, hepatocyte-specific *Acsl4* knockout (*Acsl4*^HKO^) mice were generated by crossing *Acsl4*^flox^ mice with Albumin–Cre mice (Figure S2A–D). The Gao-Binge model, which mimics the drinking patterns of patients with ALD and the early stages of human alcoholic hepatitis, was employed to induce liver injury (Figure 2A) [20]. In this model, livers from *Acsl4*^flox^ mice exhibited diffuse enlargement, a pale yellow appearance, and a greasy, soft texture compared to those from *Acsl4*^HKO^ mice (Figure 2B). Histological examination via H&E staining displayed enlarged intercellular spaces, disorganized hepatic cords, severe vacuolar steatosis, and significant infiltration of inflammatory cells in the liver sections of *Acsl4*^flox^ mice (Figure 2B). Conversely, *Acsl4*^HKO^ mice exhibited improved hepatic histology, characterized by reduced lipid vacuolation, decreased ballooning degeneration, and diminished lobular inflammation, comparable to those in the pair-fed control group (Figure 2B). While no significant differences in body weight and liver-to-body weight ratio were observed between EtOH-fed *Acsl4*^flox^ and *Acsl4*^HKO^ mice (Figure S3A,B), serum levels of ALT and AST were markedly reduced in *Acsl4*^HKO^ mice, indicating attenuated alcohol-induced liver injury (Figure 2C).

Alcoholic liver injury is driven by inflammatory responses resulting from chronic alcohol consumption [21]. Notably, hepatic infiltration of myeloid immune cells, particularly neutrophils, represents a hallmark of alcoholic liver injury [22]. Consistent with this, the hepatic immune microenvironment in *Acsl4*^HKO^ mice exhibited substantial alterations compared to *Acsl4*^flox^ controls. We observed a broad reduction in myeloid cell (CD45^+^ CD11b^+^) infiltration in the livers of *Acsl4*^HKO^ mice, with the most pronounced declines in pro-inflammatory monocytes (CD11b^+^ Ly6C^+^) and neutrophils (CD11b^+^ Ly6G^+^) (Figure 2D,E). Furthermore, in EtOH-fed *Acsl4*^flox^ mice, macrophages (CD11b^+^ F4/80^+^) and dendritic cells (CD11b^+^ CD11c^+^ MHCII^+^) were diminished, whereas these populations were restored to near-normal levels in *Acsl4*^HKO^ mice (Figure 2E). Concordantly, macrophages from *Acsl4*^HKO^ mice displayed elevated expression of anti-inflammatory M2 markers (CD11b^+^ F4/80^+^ CD206^+^) and reduced levels of M1 markers (CD11b^+^ F4/80^+^ CD86^+^) (Figure S3C). In addition, IHC staining of liver sections revealed a markedly lower expression of the neutrophil marker MPO in *Acsl4*^HKO^ mice (Figure 2F). Correspondingly, transcript levels of monocyte chemokines (*Ccl2*, *Ccl3*, and *Ccl7*) and neutrophil chemokines (*Cxcl1*, *Cxcl2*, and *Cxcl5*) were significantly downregulated in *Acsl4*^HKO^ mice (Figure 2G). In line with these results, a marked reduction in hepatic pro-inflammatory cytokines (IL-1β, TNF-α, IL-6) was observed in *Acsl4*^HKO^ mice (Figure 2H and Figure S3D). Together, these data indicate that hepatocyte-specific deletion of *Acsl4* reshapes the hepatic immune microenvironment and attenuates inflammatory responses.

### 3.3. Hepatocyte-Specific Acsl4 Ablation Alleviates Hepatic Steatosis in Alcoholic Liver Disease

**Figure 3:**
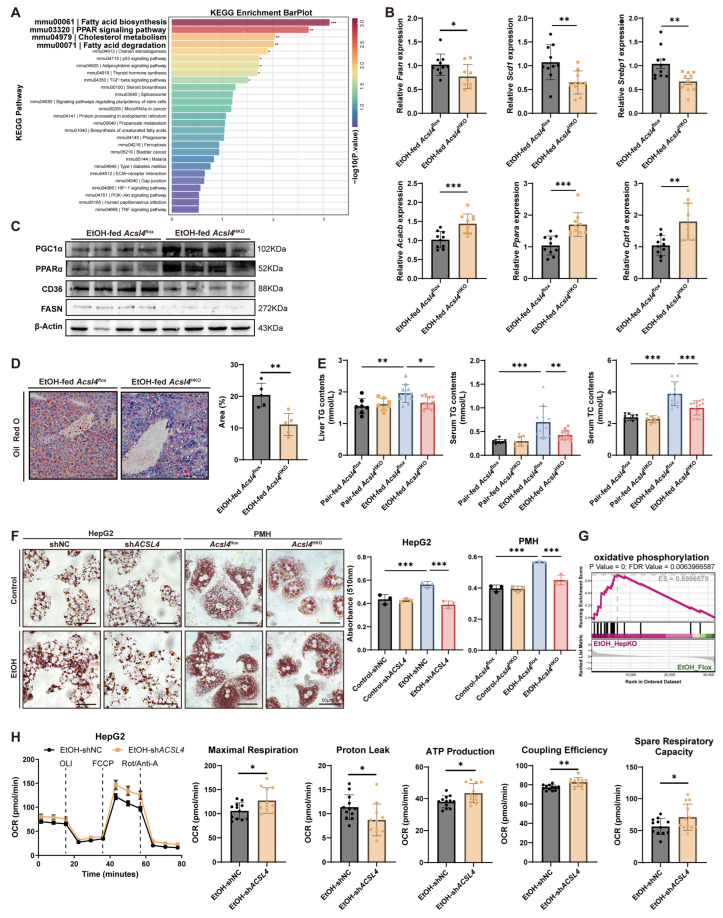
Hepatocyte-specific *Acsl4* deficiency alleviates lipid accumulation in mice with ALD. (**A**–**E**): Gao-Binge model *Acsl4*^flox^ and *Acsl4*^HKO^ mice. (**A**) KEGG enrichment analysis of DEGs from our liver RNA-seq (*n* = 5 per group). (**B**) Relative mRNA levels of genes related to fatty acid synthesis (*Fasn*, *Srebp-1c*, *Scd1*) and triglyceride oxidation (*Ppara*, *Cpt1a*, and *Acacb*) in livers (*n* = 10 per group). (**C**) Protein levels of fatty acid transporter CD36, synthesis enzyme FASN, and oxidation regulators PPARα and PGC1α in the liver. (**D**) Representative Oil Red O staining (left) and quantitative results (right) in livers from Gao-Binge model mice. Scale bar, 50 μm (*n* = 6 per group). (**E**) Hepatic TG levels (left), serum TG (middle) and T-CHO (right) levels (*n* = 10 per group). (**F**) Representative images of Oil Red O staining and quantitative results in hepatocytes treated with EtOH (100 mM, 24 h), PA (0.33 mM, 24 h), and OA (0.66 mM, 24 h). Left: HepG2 cells following transduction with shNC or sh*ACSL4* (scale bar, 50 μm; *n* = 5). Right: PMHs from *Acsl4*^flox^ and *Acsl4*^HKO^ mice (scale bar, 50 μm; *n* = 5). (**G**) GSEA enrichment plot for the oxidative phosphorylation pathway in our liver RNA-seq. (**H**) OCR profiles and key mitochondrial respiratory parameters in HepG2 cells treated with EtOH (100 mM, 24 h) following transduction with shNC or sh*ACSL4*. Data are presented as the mean ± SD. * *p* < 0.05, ** *p* < 0.01, *** *p* < 0.001 by unpaired two-tailed Student’s *t*-test (**B**,**D**,**H**) and one-way ANOVA test (**E**,**F**).

To explore the mechanisms through which ACSL4 regulates alcoholic liver injury, transcriptome RNA sequencing was performed on livers from *Acsl4*^flox^ and *Acsl4*^HKO^ mice subjected to the Gao-Binge model. KEGG enrichment analysis revealed significant enrichment of pathways associated with lipid metabolism (Figure 3A and Figure S4A). Previous studies have indicated that alcohol-induced liver injury is a progressive disorder characterized by exacerbated lipid accumulation at the onset of ALD [23]. Building on these findings, we further examined the role of ACSL4 in lipid metabolism during ALD progression.

In *Acsl4*^HKO^ mice, the expression of genes related to fatty acid synthesis (*Fasn*, *Scd1*, *Srebp1* and *Cd36*) was downregulated, while the expression of genes involved in fatty acid oxidation (*Ppara*, *Pgc1a*, *Cpt1a*, and *Acacb*) was upregulated (Figure 3B,C and Figure S4B). Consistent with these expression changes, *Acsl4*^HKO^ mice exhibited markedly reduced hepatic lipid accumulation compared with controls, as visualized by Oil Red O staining (Figure 3D). Furthermore, levels of T-CHO, TG, and hepatic TG were significantly lower in *Acsl4*^HKO^ mice than in littermate controls (Figure 3E). ACSL4 ablation in HepG2 cells (Figure S2E,F), as well as in PMHs isolated from *Acsl4*^HKO^ mice, significantly attenuated alcohol-induced lipid accumulation following treatment with free fatty acids (palmitic acid, PA, 0.33 mM; oleic acid, OA, 0.66 mM) and 24 h ethanol exposure (Figure 3F).

Given the notable enrichment of pathways related to oxidative phosphorylation (Figure 3G), we further investigated the role of ACSL4 in fatty acid oxidation and mitochondrial respiration following alcohol exposure. To this end, we performed Seahorse mitochondrial stress assays using ACSL4-knockdown HepG2 cells following ethanol treatment. The results demonstrated that ACSL4 knockdown enhanced the mitochondrial OCR, as evidenced by increased maximal respiration, ATP production, coupling efficiency, and spare respiratory capacity, along with decreased proton leak (Figure 3H and Figure S4C). These findings collectively indicate that hepatocyte-specific ACSL4 deletion attenuates alcohol-induced hepatic lipid dysregulation.

### 3.4. ACSL4 Regulates Alcohol-Induced Oxidative Stress Through Metabolite γ-GC

**Figure 4:**
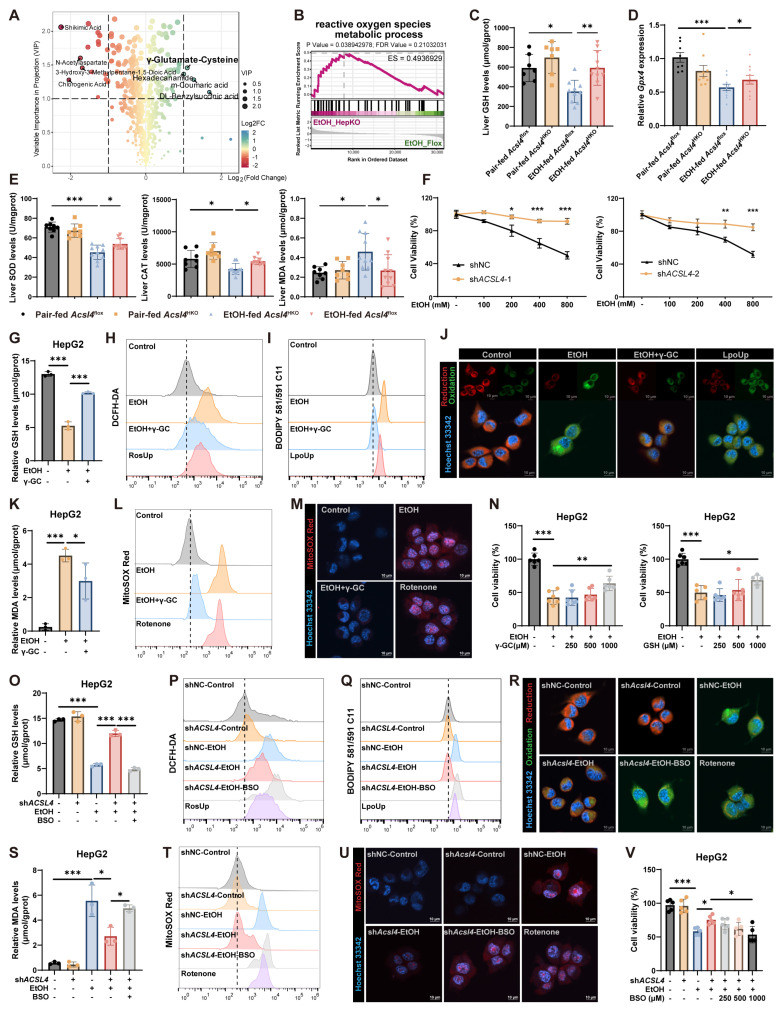
Hepatocyte-specific *Acsl4* deletion mitigates oxidative stress in alcohol-induced liver injury. (**A**) Volcano plot of differential metabolites in livers of *Acsl4*^flox^ and *Acsl4*^HKO^ mice. (**B**) GSEA enrichment plot for the reactive oxygen species metabolic process in our liver RNA-seq. (**C**) Hepatic GSH levels in Gao-Binge model *Acsl4*^flox^ and *Acsl4*^HKO^ mice (Pair-fed, *n* = 8; EtOH-fed, *n* = 10). (**D**) Relative mRNA levels of *Gpx4* in livers from Gao-Binge model *Acsl4*^flox^ and *Acsl4*^HKO^ mice (Pair-fed, *n* = 8; EtOH-fed, *n* = 10). (**E**) Hepatic SOD, CAT, MDA levels in Gao-Binge model *Acsl4*^flox^ and *Acsl4*^HKO^ mice (Pair-fed, *n* = 8; EtOH-fed, *n* = 10). (**F**) Cell viability of HepG2 cells treated with EtOH (100–800 mM, 24 h) following transduction with shNC, sh*ACSL4*-1, sh*ACSL4*-2. (**G**) Cellular GSH levels in HepG2 cells treated with EtOH (400 mM, 24 h) and γ-GC (500 μM, 24 h). (**H**) DCFH-DA fluorescence (ROS levels) in HepG2 cells treated with EtOH (400 mM, 24 h) and γ-GC (500 μM, 24 h). RosUp/H_2_O_2_ was used as a positive control. (**I**,**J**) BODIPY 581/591 C11 fluorescence (lipid peroxidation levels) in HepG2 ((**I**), flow cytometry) and AML12 ((**J**), confocal imaging) cells treated with EtOH (400 mM/100 mM, 24 h) and γ-GC (500 μM, 24 h). Scale bar, 10 μm. LpoUp/RSL3 was used as a positive control. (**K**) Cellular MDA levels in HepG2 cells treated with EtOH (400 mM, 24 h) and γ-GC (500 μM, 24 h). (**L**–**M**) MitoSOX Red fluorescence (mitochondrial superoxide levels) in HepG2 ((**L**), flow cytometry) and AML12 ((**M**), confocal imaging) cells treated with EtOH (400 mM/100 mM, 24 h) and γ-GC (500 μM, 24 h). Scale bar, 10 μm. Rotenone served as a positive control. (**N**) Cell viability of HepG2 cells treated with EtOH (400 mM, 24 h), γ-GC (125, 250, 500 μM, 24 h, left), GSH (250, 500, 1000 μM, 24 h, right). (**O**) Cellular GSH levels in HepG2 cells treated with EtOH (400 mM, 24 h) and BSO (1 mM, 24 h) following transduction with shNC or sh*ACSL4*. (**P**) DCFH-DA fluorescence (ROS levels) in HepG2 cells treated with EtOH (400 mM, 24 h) and BSO (1 mM, 24 h) following transduction with shNC or sh*ACSL4*. RosUp/H_2_O_2_ was used as a positive control. (**Q**,**R**) BODIPY 581/591 C11 fluorescence (lipid peroxidation levels) in HepG2 ((**Q**), flow cytometry) and AML12 ((**R**), confocal imaging) cells treated with EtOH (400 mM, 24 h) and BSO (1 mM, 24 h) following transduction with shNC or sh*Acsl4*. Scale bar, 10 μm. LpoUp/RSL3 was used as a positive control. (**S**) Cellular MDA levels in HepG2 cells treated with EtOH (400 mM, 24 h) and BSO (1 mM, 24 h) following transduction with shNC or sh*ACSL4*. (**T**,**U**) MitoSOX Red fluorescence (mitochondrial superoxide levels) in HepG2 ((**T**), flow cytometry) and AML12 ((**U**), confocal imaging) cells treated with EtOH (400 mM/100 mM, 24 h) and BSO (1 mM, 24 h) following transduction with shNC or sh*ACSL4.* Scale bar, 10 μm. Rotenone served as a positive control. (**V**) Cell viability of HepG2 cells treated with EtOH (400 mM, 24 h) and BSO (1 mM, 24 h) following transduction with shNC or sh*ACSL4*. Data are presented as the mean ± SD. * *p* < 0.05, ** *p* < 0.01, *** *p* < 0.001 by unpaired two-tailed Student’s *t*-test (**F**) and one-way ANOVA test (**C**–**E**,**G**,**K**,**N**,**O**,**S**,**V**).

Given the significant impact of ACSL4 on the hepatic metabolic environment, we conducted metabolomic assay on *Acsl4*^HKO^ and their control littermate mice. Results revealed that γ-GC was one of the most markedly upregulated metabolites (Figure 4A). As is well-established, in addition to inducing dysregulated lipid metabolism, excessive alcohol consumption up-regulates the expression of CYP2E1, leading to the accumulation of ROS and ultimately culminating in hepatic oxidative stress [24]. Oxidative stress induced by alcohol metabolism depletes GSH, thereby reducing GPX4 activity and impairing the clearance of lipid peroxides. γ-GC, a key intermediate in the GSH biosynthetic pathway, participates in ROS scavenging and assists GSH in clearing LPOs. Functioning as a cellular brake against peroxidation and ferroptosis, γ-GC plays an essential role in intracellular antioxidant defense and the regulation of redox homeostasis [25]. Gene Set Enrichment Analysis (GSEA) confirmed significant enrichment in pathways related to reactive oxygen species, glutamine metabolism, and glutathione peroxidase activity (Figure 4B and Figure S5A). Consistent with this, hepatocyte-specific knockdown of *Acsl4* in the Gao-Binge model significantly elevated GSH levels and upregulated *Gpx4* expression (Figure 4C,D). Concurrently, *Acsl4*^HKO^ mice displayed not only enhanced activity of key antioxidant enzymes (CAT and SOD) but also markedly reduced levels of lipid peroxidation products (e.g., MDA, Figure 4E).

According to the canonical theory, ACSL4 incorporates PUFAs into membrane phospholipids, thereby markedly increasing membrane susceptibility to lipid peroxidation and serving as a key molecular determinant of ferroptosis [17]. However, in cells treated with ethanol alone (without exogenous PUFA supplementation), ACSL4 knockdown conferred increased resistance to ethanol-induced cell death, as assessed by cell viability assays and morphological observation (Figure 4F and Figure S5B,C). Concurrently, LDH release was significantly attenuated following ACSL4 inhibition, indicating reduced plasma membrane rupture and cell death (Figure S5D). These results indicated that ACSL4 knockdown may directly attenuate ferroptosis by inhibiting ROS production and halting lipid peroxidation, an effect consistent with the physiological function of γ-GC.

In subsequent in vitro experiments, γ-GC supplementation alleviated cellular oxidative stress by increasing GSH levels (Figure 4G). The elevated GSH effectively attenuated alcohol-induced ROS accumulation, as measured by DCFH-DA fluorescence (Figure 4H and Figure S5E). Consequently, the reduction in ROS led to decreased lipid peroxidation, assessed using the BODIPY 581/591 C11 probe (Figure 4I,J and Figure S5F,G), and was further reflected by a reduction in MDA content (Figure 4K). Given that ACSL4 knockdown significantly reduced total ROS and lipid peroxidation, and in light of its effects on mitochondrial respiration, we hypothesized that mitochondria are the primary source of this oxidative stress. To test this, we measured mitochondrial superoxide using MitoSOX Red fluorescence probe. The results showed that γ-GC treatment significantly reduced the proportion of MitoSOX Red-positive cells (Figure 4L,M and Figure S5H,I). Furthermore, supplementation with either γ-GC or GSH significantly mitigated alcohol-induced cytotoxicity (Figure 4N). These findings suggest that, ACSL4 knockdown may reduce sensitivity to ferroptosis not only by remodeling membrane phospholipid composition but also through directly enhancing bioactive peptide-mediated antioxidant capacity.

DL-Buthionine-(S,R)-sulfoximine (BSO), a specific and potent inhibitor of γ-GC synthesis, effectively suppresses GSH production. BSO supplementation reversed the protective effects in ACSL4-knockdown cells, as evidenced by increased oxidative stress markers (Figure 4O–U and Figure S5J–N) and by the resensitization of these cells to ethanol- and erastin-induced cytotoxicity (Figure 4V and Figure S5O). These findings collectively demonstrate that γ-GC serves as a key metabolic mediator downstream of ACSL4, and that ACSL4 knockdown attenuates alcohol-induced lipid peroxidation through the antioxidant γ-GC-GSH axis.

### 3.5. γ-GC Binds to PTP4A1 and Suppresses the Downstream MAPK-NF-κB Signaling Pathway

**Figure 5:**
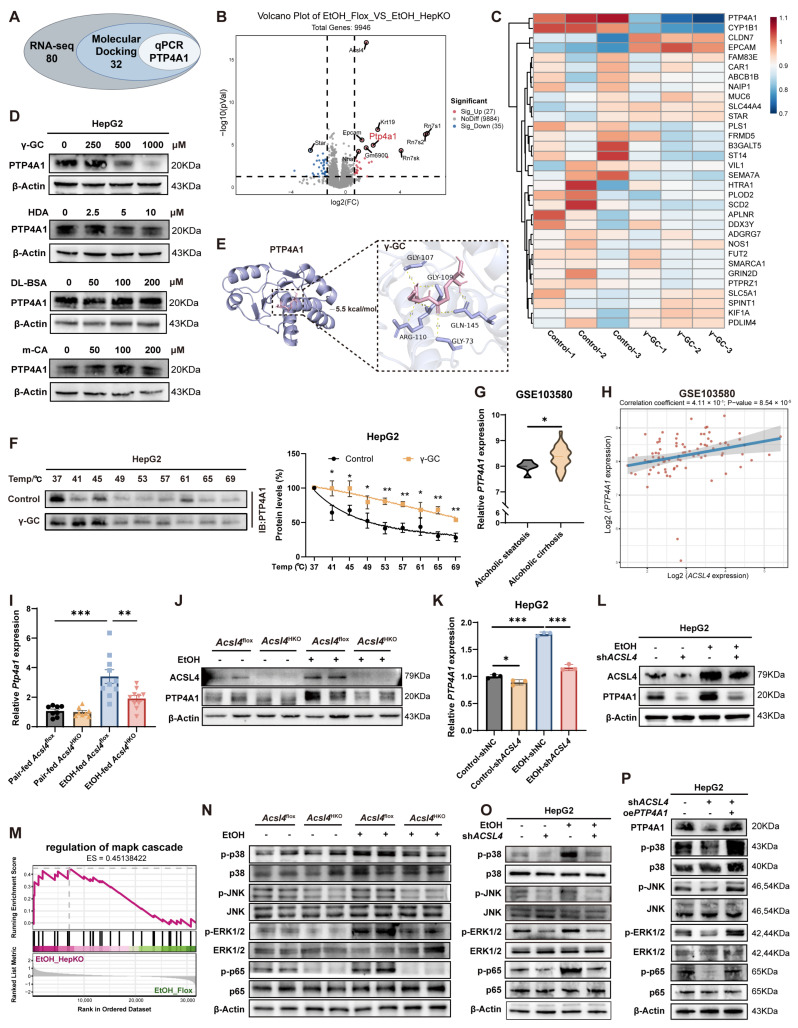
ACSL4 deficiency attenuates MAPK signaling via the interaction between γ-GC and PTP4A1. (**A**) Venn diagram showing the overlapping candidate genes identified from transcriptomic analysis, molecular docking, and qPCR validation. (**B**) Volcano plots of DEGs from our liver RNA-seq (*n* = 5). (**C**) Heatmap of mRNA levels of high-affinity DEGs identified by molecular docking following γ-GC treatment. (**D**) Protein levels of PTP4A1 in HepG2 cells treated with γ-GC (250, 500, 1000 μM), HDA (2.5, 5, 10 μM), m-CA (50, 100, 200 μM), and DL-BSA (50, 100, 200 μM) for 24 h. (**E**) Molecular docking model of the PTP4A1-γ-GC complex. (**F**) Thermal stability analysis of PTP4A1 in HepG2 cells treated with γ-GC (1000 μM, 2 h). Representative immunoblots (left) and quantification (right). (**G**) Relative expression levels of PTP4A1 in liver samples from GSE103580 (Alcoholic steatosis, *n* = 6; Alcoholic cirrhosis, *n* = 67). (**H**) Correlation between ACSL4 and PTP4A1 expression in liver samples from GSE103580, determined by Pearson correlation test. (**I**,**J**) Relative mRNA (**I**) and protein (**J**) levels of PTP4A1 in livers from Gao-Binge model *Acsl4*^flox^ and *Acsl4*^HKO^ mice (Pair-fed, *n* = 8; EtOH-fed, *n* = 10). (**K**,**L**) Relative mRNA (**K**) and protein (**L**) levels of PTP4A1 in HepG2 cells treated with EtOH (100 mM, 24 h) following transduction with shNC or sh*ACSL4.* (**M**) GSEA enrichment plot for the regulation of MAPK cascade in our liver RNA-seq. (**N**) Protein levels of phospho- and total MAPK/NF-κB pathway components (p38, JNK, ERK1/2, and p65) in livers from Gao-Binge model *Acsl4*^flox^ and *Acsl4*^HKO^ mice. (**O**) Protein levels of MAPK/NF-κB pathway in HepG2 cells treated with EtOH (100 mM, 24 h) following transduction with shNC or sh*ACSL4.* (**P**) Protein levels of MAPK/NF-κB pathway in HepG2 cells following transduction with shNC or sh*ACSL4*, with or without pcDNA3.1-*PTP4A1.* Data are represented as the mean ± SD. * *p* < 0.05, ** *p* < 0.01, *** *p* < 0.001 by unpaired two-tailed Student’s *t*-test (**G**), one-way ANOVA test (**I**,**K**), two-way ANOVA test (**F**).

To identify downstream effectors of γ-GC in ALD, we performed transcriptomic sequencing on livers from *Acsl4*^HKO^ and *Acsl4*^flox^ mice and analyzed the resulting DEGs (Figure 5A,B). Molecular docking was then carried out between γ-GC and the proteins encoded by the most significantly dysregulated genes. This computational screening identified 32 candidate proteins with high predicted binding affinity (docking score < −5.0 kcal/mol) for γ-GC (Figure 5A and Figure S6A). Subsequent transcriptional profiling following γ-GC treatment identified PTP4A1 as the most strongly regulated candidate among these targets (Figure 5A,C).

We next evaluated metabolites upregulated in *Acsl4*^HKO^ mice—including γ-GC, hexadecanamide (HDA), m-coumaric acid (m-CA), and DL-benzylsuccinic acid (DL-BSA)—for their ability to modulate PTP4A1. Analysis of both mRNA and protein levels confirmed that only γ-GC suppressed PTP4A1 expression (Figure 5D and Figure S6B). Molecular docking revealed a high binding affinity and key hydrogen bonding interactions between PTP4A1 and γ-GC (Figure 5E and Figure S6C). However, co-immunoprecipitation (Co-IP) experiments detected no direct protein–protein interaction between ACSL4 and PTP4A1 (Figure S6D). We then performed the CETSA to evaluate target engagement in a cellular context. Notably, γ-GC treatment markedly altered the thermal stability of PTP4A1 (Figure 5F), indicating direct ligand-induced stabilization.

Analysis of a public clinical database revealed that hepatic PTP4A1 expression was significantly elevated in patients with alcohol-associated liver disease (Figure 5G). Moreover, PTP4A1 levels exhibited a strong positive correlation with ACSL4 expression (Figure 5H). Consistent with this clinical observation, in the Gao-Binge mouse model, ethanol-fed *Acsl4*^HKO^ mice showed marked downregulation of hepatic *Ptp4a1* expression compared to EtOH-fed *Acsl4*^flox^ littermates (Figure 5I,J and Figure S6E). Consistently, ACSL4 knockdown in vitro resulted in reduced PTP4A1 expression (Figure 5K,L and Figure S6F,G), further supporting a regulatory relationship between ACSL4 and PTP4A1.

Protein tyrosine phosphatases (PTPs), including PTP4A1, play crucial roles in cellular signaling by modulating tyrosine phosphorylation [26]. PTP4A1 has been implicated in the regulation of ERK1/2 [27], p53 [28], and PTEN/PI3K/Akt [29] signaling pathways. Of note, the mitogen-activated protein kinase (MAPK) signaling pathway, comprising JNK, p38, and ERK1/2, mediates inflammatory cytokine production [30,31]. Our transcriptomic data indicated significant enrichment of MAPK-related pathways (Figure 5M and Figure S6H). Consistent with this, ACSL4 inhibition reduced phosphorylation of p38, JNK, and ERK1/2, leading to suppressed nuclear factor kappa-B (NF-κB) activation both in vivo and in vitro (Figure 5N,O and Figure S6I). Crucially, PTP4A1 overexpression restored phosphorylation levels of these kinases (Figure 5P and Figure S6I), confirming its role as a key mediator of ACSL4-regulated inflammation.

Collectively, these findings demonstrate that γ-GC, a metabolite liberated following ACSL4 knockdown, directly binds to PTP4A1 and suppresses its expression, thereby attenuating the MAPK-NF-κB-mediated inflammatory cascade.

### 3.6. The PTP4A1 Inhibitor JMS-053 Protects Against Alcohol-Induced Liver Injury by Impeding the MAPK Signaling Pathway

**Figure 6:**
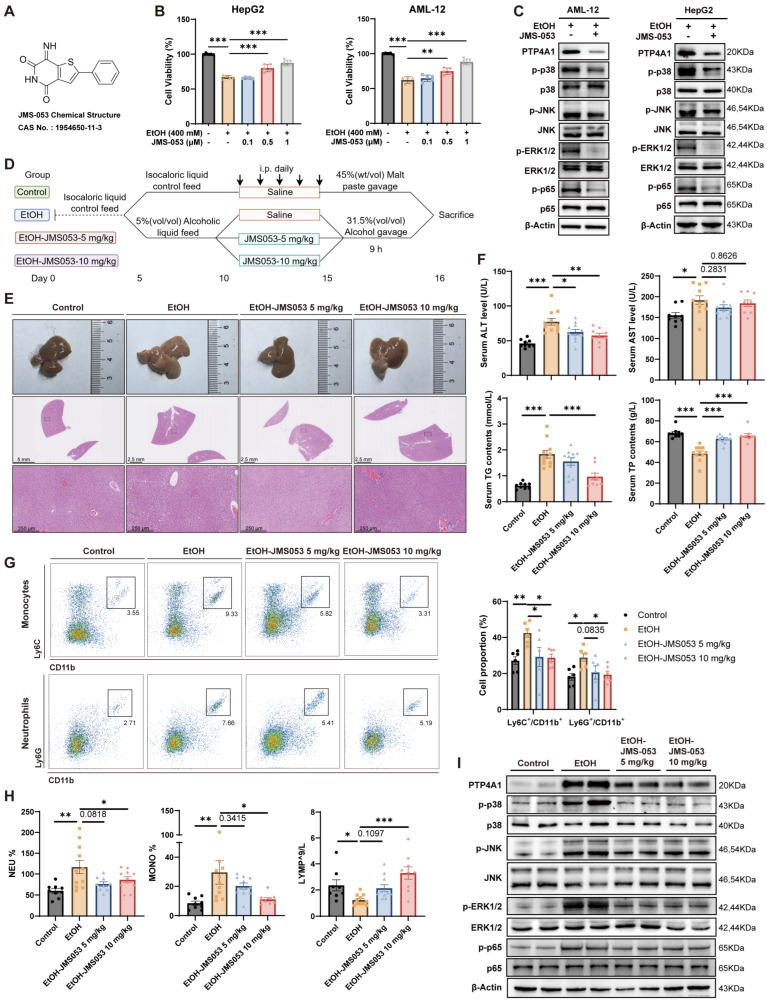
Pharmacological PTP4A1 inhibition improves pathological characteristics of alcoholic injury by impeding the MAPK/NF-κB signaling pathway. (**A**) Chemical structure of JMS-053. (**B**) Cell viability of HepG2 and AML12 cells treated with EtOH (400 mM, 24 h) and JMS-053 (0.1, 0.5, 1 μM, 24 h). (**C**) Protein levels of MAPK/NF-κB pathway components in HepG2 and AML12 cells treated with EtOH (400 mM, 24 h) and JMS-053 (1 μM, 24 h). (**D**) Schematic diagram of the JMS-053-treated Gao-Binge model mice. (**E**) Representative images of liver morphology and H&E staining from JMS-053-treated Gao-Binge model mice. Scale bar, 2.5 mm (gross morphology) and 200 μm (H&E); *n* = 6 per group. (**F**) Serum ALT, AST, TG, and TP levels in JMS-053-treated Gao-Binge model mice (Control, *n* = 9; EtOH, *n* = 11; JMS-053 5 mg/kg, *n* = 11; JMS-053 10 mg/kg, *n* = 9). (**G**) Flow cytometric analysis of hepatic myeloid cells from JMS-053-treated Gao-Binge model mice. Representative density plots (upper) and quantitative analysis (bottom) showing the expression of Ly6C^+^CD11b^+^ and Ly6G^+^CD11b^+^ cells. Cells were gated on live, singlet, CD45^+^ cells. (**H**) CBC parameters: neutrophil (NEU%) and monocyte (MONO%) percentages; lymphocyte (LYMP) counts (×10^9^/L) in JMS-053-treated Gao-Binge model mice. (**I**) Protein levels of MAPK/NF-κB pathway components in livers from JMS-053-treated Gao-Binge model mice. Data are represented as the mean ± SD, * *p* < 0.05, ** *p* < 0.01, *** *p* < 0.001 by one-way ANOVA test (**B**,**F**–**H**).

We further investigated the role of the PTP4A1 inhibitor JMS-053 in alcohol-associated liver injury (Figure 6A). In vitro, JMS-053 effectively mitigated alcohol-induced cell death in a dose-dependent manner (Figure 6B) and attenuated phosphorylation of key components within the MAPK-NF-κB pathway (Figure 6C and Figure S7A).

To assess its efficacy in vivo, we utilized the Gao-Binge model and administered JMS-053 intraperitoneally at doses of 5 mg/kg and 10 mg/kg (Figure 6D). Treatment was initiated on day 10, upon confirmation of mild liver injury (Figure S7B). Although no significant alteration in body weight or liver-to-body weight ratio was observed in this short-term intervention (Figure S7C,D), both the gross appearance and histological examinations revealed improved liver pathology in JMS-053-treated mice (Figure 6E). JMS-053 administration effectively reduced serum ALT levels, indicating mitigated liver injury (Figure 6F). Furthermore, JMS-053 significantly decreased TG levels and increased total protein (TP) levels (Figure 6F). Flow cytometry and CBC analysis demonstrated a pronounced reduction in the infiltration of inflammatory monocytes and neutrophils after treatment, while lymphocyte counts were restored to levels comparable to the control group (Figure 6G,H).

Furthermore, we evaluated the safety profile of JMS-053. Serum biochemistry and CBC results indicated that, compared to the model group, JMS-053 did not impose additional burden on the coagulation system, liver function, or renal function (Figure S7E,F). Toxicological evaluation via H&E staining of multiple organs (heart, spleen, lung, and kidney) revealed no additional drug-induced toxicity compared to the model group (Figure S8A). Notably, pathological manifestations in the alcohol model group—including dilation of Bowman’s capsule containing eosinophilic amorphous material, shrinkage of glomerular tufts, and the presence of eosinophilic amorphous material within renal tubules—were also attenuated by JMS-053 treatment (Figure S8B).

Consistent with our mechanistic findings, JMS-053 treatment suppressed activation of the MAPK-NF-κB pathway in liver tissues (Figure 6I and Figure S7G). Taken together, these results demonstrate that JMS-053, a selective inhibitor of PTP4A1, attenuates disease progression and inflammatory signaling in alcohol-related liver disease, mirroring the protective effects of genetic *Acsl4* ablation.

### 3.7. Targeting ACSL4 with Dronedarone Effectively Alleviates the Progression of Alcoholic Liver Disease

**Figure 7:**
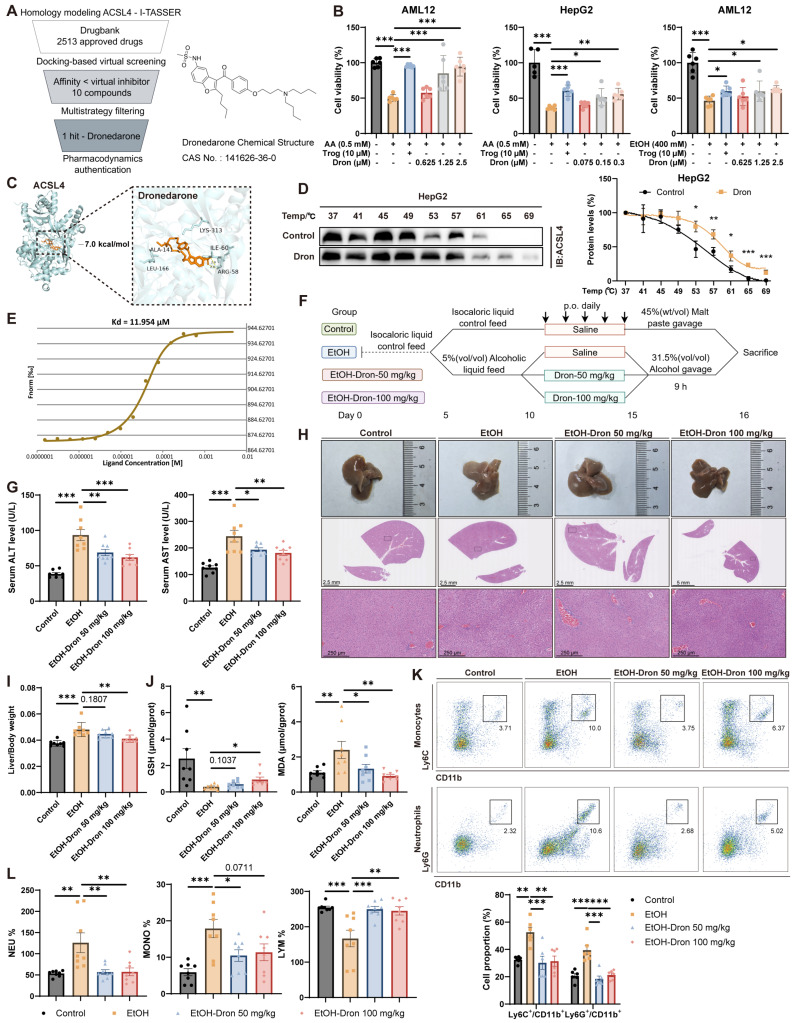
Pharmacological inhibition of ACSL4 with dronedarone markedly attenuated the progression of ALD. (**A**) Screening pipeline for ACSL4-targeted compounds (left) and chemical structure of dronedarone (right). (**B**) Cell viability of HepG2 and AML12 cells treated with AA (0.2 mM, 24 h) or EtOH (400 mM, 24 h), and Dron (24 h). (**C**) Molecular docking model of the Dron-ACSL4 complex. (**D**) Thermal stability analysis of ACSL4 in HepG2 cells treated with Dron (20 μM, 3 h). Representative immunoblots (left) and quantification (right). (**E**) Dose–response curve with Dron-ACSL4 binding affinity (Kd = 11.954 μM) derived from the MST data. (**F**) Schematic diagram of the Dron-treated Gao-Binge model mice. (**G**) Serum ALT, AST levels in Dron-treated Gao-Binge model mice (*n* = 8 per group). (**H**) Representative liver morphology and H&E staining from Dron-treated Gao-Binge model mice Scale bar, 2.5 mm (gross morphology) and 200 μm (H&E); *n* = 6 per group. (**I**) Liver/body weight ratio in Dron-treated Gao-Binge model mice. (**J**) Hepatic GSH and MDA levels in Dron-treated Gao-Binge model mice. (**K**) Flow cytometric analysis of hepatic myeloid cells from Dron-treated Gao-Binge model mice. Representative density plots (upper) and quantitative analysis (bottom) showing the expression of Ly6C^+^CD11b^+^ and Ly6G^+^CD11b^+^ cells. Cells were gated on live, singlet, CD45^+^ cells. (**L**) CBC parameters: neutrophil (NEU%), monocyte (MONO%), and lymphocyte (LYM%) percentages in Dron-treated Gao-Binge model mice. Data are represented as the mean ± SD, * *p* < 0.05, ** *p* < 0.01, *** *p* < 0.001 by one-way ANOVA test (**B**,**G**,**I**–**L**), two-way ANOVA test (**D**).

Despite the established role of ACSL4 in disease pathogenesis, the development of targeted inhibitors remains limited. To develop novel small-molecule inhibitors targeting ACSL4, we performed a structure-based virtual screening of an FDA-approved drug library containing 2513 compounds using machine learning and deep learning-assisted molecular docking (Figure 7A). Ten candidate compounds exhibiting higher binding affinity than the virtual inhibitor were selected for further experimental validation (Figure S9A). Given the role of ACSL4 in promoting AA incorporation and lipid peroxidation, we assessed the ability of candidate compounds to rescue AA-induced cell death in HepG2 and AML12 cells. Using troglitazone and the ferroptosis inhibitor Fer-1 as positive controls, we observed that dronedarone conferred protection against AA-induced cytotoxicity comparable to that of both positive controls (Figure 7B, Figures S9B and S10A,B). This protective effect was consistently reproduced in alcohol-induced cell death models (Figure 7B). Molecular docking with AutoDock Vina suggested a potential binding mode between dronedarone and ACSL4 (Figure 7C). This interaction was further validated by CETSA and MST, confirming high-affinity binding (Figure 7D,E and Figure S9C).

We next evaluated the therapeutic efficacy of dronedarone in vivo using the Gao-Binge model, administering dronedarone at 50 mg/kg and 100 mg/kg (Figure 7F). Dron treatment significantly reduced serum ALT and AST levels, indicating attenuated liver injury (Figure 7G). Macroscopic and histopathological evaluation of liver tissues revealed substantial improvement in dronedarone-treated groups (Figure 7H). Although body weights did not differ significantly among EtOH-fed groups (Figure S11A), the liver-to-body weight ratio was markedly decreased in the 100 mg/kg dronedarone-treated group (Figure 7I). Concurrently, dronedarone treatment led to a marked elevation in GSH levels and a reduction in MDA, underscoring its efficacy in attenuating oxidative stress and lipid peroxidation (Figure 7J). Flow cytometry and CBC results further revealed decreased infiltration of myeloid-derived inflammatory cells and an expansion of lymphocyte populations in dronedarone-administered groups (Figure 7K,L).

In addition, dronedarone administration showed no overt hepatotoxicity, nephrotoxicity, or hematological toxicity, as evidenced by serum biochemistry and CBC results, indicating a favorable safety profile (Figure S11B,C). Histopathological examination of extrahepatic organs (heart, spleen, lung, and kidney) revealed no evidence of drug-related toxicity or pathological alterations following treatment (Figure S12A). Notably, alcohol-induced renal abnormalities, such as Bowman’s capsule dilation with eosinophilic material accumulation and glomerular tuft shrinkage, were also alleviated by dronedarone treatment (Figure S12B).

Together, these results indicate that dronedarone attenuates alcoholic liver disease through targeting ACSL4, demonstrating favorable efficacy and safety profiles that support its therapeutic potential.

## 4. Discussion

Excessive alcohol consumption represents a major global health challenge, contributing to significant morbidity and mortality that impose considerable medical, economic, and social burdens [32]. Alcohol-related liver disease is responsible for an estimated 5.1% of the global burden of disease and injury, and alcohol consumption accounts for 5.3% of all deaths worldwide, positioning ALD among the leading causes of mortality [33]. The mechanisms underlying alcoholic liver injury are intricate and multifaceted. Although recent clinical investigations have indicated that the combination of N-acetylcysteine and corticosteroids offers superior recovery outcomes in ALD patients [34], approved pharmacotherapies specifically targeting ALD remain limited [35].

Mounting evidence indicates that chronic ethanol consumption disrupts hepatic lipid metabolism and promotes oxidative stress. Adducts arising from lipid peroxidation, glutathione depletion, and mitochondrial dysfunction elicit immune responses, leading to hepatocyte injury and death [36]. Ferroptosis, an iron-dependent and non-apoptotic cell death driven by lipid peroxidation, is closely linked to oxidative stress and dysregulated cysteine metabolism [33]. It has been widely implicated in the pathogenesis of ALD [37,38]. ACSL4, a key biomarker and regulator of ferroptosis, dictates cell susceptibility to this form of cell death through lipid remodeling [13]. It serves not only as a sensitive indicator of ferroptosis but also as a critical mediator of lipid metabolic reprogramming [14].

In this study, hepatocyte-specific knockdown of *Acsl4* alleviated alcohol-induced liver pathology in the Gao-Binge mouse model of ALD, attenuating both metabolic dysregulation and inflammatory progression. Mechanistically, ACSL4 silencing reduced alcohol-induced oxidative stress via the release of the metabolite γ-GC. Furthermore, γ-GC targeted the downstream protein PTP4A1, and the suppressed PTP4A1 inhibited the MAPK-NF-κB inflammatory cascade. Pharmacological inhibition of PTP4A1 with JMS-053 attenuated alcohol-induced liver injury in vivo, phenocopying the protective effects of *Acsl4* knockdown. Through structure-based drug discovery—including homology modeling, molecular docking, and pharmacological screening—we identified dronedarone as a promising ACSL4-targeting compound. Treatment with dronedarone significantly attenuated the progression of alcoholic liver disease in experimental models without inducing additional toxicity. Collectively, these findings underscore ACSL4 as a promising therapeutic target for ALD management.

Ferroptosis is modulated by diverse metabolic pathways, including dysregulation of the antioxidant system (particularly involving amino acids and peptides), disordered iron homeostasis, and the accumulation of lipid peroxides. ACSL4 has been widely recognized to catalyze the conversion of PUFAs into PUFA-CoAs, which are subsequently esterified by LPCAT3 and oxidized by lipoxygenases to generate lipid peroxides. The excessive accumulation of these peroxides leads to irreversible damage to cellular membranes, ultimately resulting in cell death. In this study, we uncovered a previously unrecognized role of ACSL4 in directly modulating oxidative stress through the bioactive peptide-mediated antioxidant system in alcoholic liver disease. Unlike metabolic liver disorders, where lipid accumulation drives PUFA overload and subsequent peroxidation, ethanol metabolism—primarily mediated by CYP2E1—generates substantial ROS. This depletes antioxidants such as GSH, leading to severe oxidative stress. The relative deficiency of PUFAs in this context suggests that ACSL4 may not solely dictate ferroptosis sensitivity via PUFA metabolism. Furthermore, our metabolomic analyses revealed that *Acsl4* knockdown did not significantly alter the composition of membrane phospholipids, contrasting with observations in high-fat models. This indicates that ACSL4 may exert antioxidant effects through alternative pathways in the absence of a lipid-rich environment. Our findings point to a peptide-mediated antioxidant pathway as a crucial alternative mechanism in alcohol-induced injury.

Our metabolomic profiling uncovered the antioxidant bioactive peptide γ-GC, which serves as a critical precursor for GSH synthesis. γ-GC has been demonstrated to attenuate insulin resistance and reduce the incidence of diabetic complications by attenuating cellular senescence and suppressing ectopic lipid deposition [39]. These findings indicate the therapeutic potential of γ-GC for alleviating pathological manifestations in liver disease. In summary, our study elucidated a novel mechanism through which ACSL4 regulates hepatic redox homeostasis via γ-GC, suggesting a potential role of ACSL4 in the direct regulation of the antioxidant system. Alcohol consumption promotes hepatic lipogenesis while suppressing fatty acid oxidation, resulting in the accumulation of PUFAs, which serve as the “fuel” for ferroptosis. Concurrently, ethanol metabolism generates abundant ROS, acting as the “spark” that ignites lipid peroxidation. Alcohol-induced oxidative stress further disables the cellular “firefighting system” by depleting GSH, thereby impairing GPX4 activity and compromising the clearance of lipid peroxides. ACSL4 knockdown counteracts this process in a dual manner: it reduces the availability of PUFA substrates, thereby limiting the “fuel”, and enhances γ-GC production, which diminishes ROS generation (the “spark”) and restores the “firefighting system” by increasing GSH levels and GPX4 activity. Together, these actions collectively suppress the initiation and progression of ferroptosis.

Moreover, γ-GC has been demonstrated to exert protective effects against inflammation in various disease contexts. For instance, it mitigates TNBS-induced inflammatory bowel disease via modulating macrophage polarization [40], and reduces microglial neuroinflammation triggered by amyloid-β oligomers through NF-κB pathway inhibition [41]. In this study, we demonstrated that ACSL4 modulates the pro-inflammatory cascade through γ-GC-mediated action on PTP4A1. PTPs constitute a large family of enzymes critical for the activation of signaling pathways that determine cell fate [26]. Dysregulation of PTP activity, leading to aberrant tyrosine phosphorylation, has been implicated in various diseases, including metabolic disorders and cancer [28,29,42,43]. The human phosphatase of regenerating liver (PRL) family is composed of three members (PTP4A1, PTP4A2, and PTP4A3) [44,45]. Notably, tissue expression profiling indicates that PTP4A1 is highly expressed in the liver, while PTP4A2 and PTP4A3 are virtually undetectable in this organ [45]. As a dual-specificity phosphatase, PTP4A1 plays essential roles in cell development and tissue regeneration [46]. Based on its structural motifs, PTP4A1 interacts with plasma membrane phospholipids and modulates cellular sensitivity to oxidative stress [47,48,49]. It has also been identified as a candidate gene for alcohol dependence in multiple genome-wide association studies and is functionally linked to other genes implicated in alcohol addiction [50]. Nonetheless, the role of PTP4A1 in alcoholic liver injury remains to be elucidated. This study establishes PTP4A1 as a critical node linking oxidative stress to inflammatory cascades in ALD. JMS-053, a potent and selective reversible inhibitor of PTP4A, exerts protective effects against alcoholic liver injury. Our results demonstrate that JMS-053 enhances cellular resistance to alcohol-induced injury and attenuates inflammation through suppression of the MAPK-NF-κB signaling pathway.

In addition, our results demonstrated that ACSL4 inhibition enhanced hepatic alcohol metabolism, as evidenced by significant alterations in the expression of key metabolic enzymes including *Adh1*, *Aldh2*, and *Cyp2e1* (Figure S3E). This observation was further corroborated by GSEA, which revealed significant enrichment of gene sets associated with alcohol dehydrogenase (NAD+) and aldehyde dehydrogenase (NAD+) activities (Figure S3F). While our study primarily establishes that ACSL4 attenuates ALD progression through mitigating lipid peroxidation and inflammatory signaling, these data also suggest a potential role of ACSL4 in modulating alcohol metabolic flux and oxidative stress handling. However, the exact mechanistic link between ACSL4 and ethanol-metabolizing enzymes remains unclear and warrants further investigation.

This study has several limitations. First, our key findings are primarily derived from the Gao-Binge mouse model, which, although widely used, may not fully capture the clinical heterogeneity and pathological complexity of human ALD. Validation in additional ALD models, such as chronic feeding or genetic diversity panels, will help generalize our findings. Second, the precise mechanism by which ACSL4 deletion leads to increased γ-GC production remains incompletely defined. Specifically, whether ACSL4 directly modulates glutamate–cysteine ligase activity or affects the intracellular availability of its substrates warrants further experimental validation. Addressing these points in future studies will enhance the clinical relevance and mechanistic depth of our conclusions.

In summary, this study delineates the pathogenic role and mechanistic basis of ACSL4 in accelerating the progression of ALD and unveils novel perspectives on its regulatory function in ferroptosis. We propose the novel repurposing of dronedarone for targeting ACSL4, highlighting a potential avenue for the clinical management of ALD.

## Data Availability

RNA-seq data have been deposited at Sequence Read Archive (SRA) as PRJNA1320650 (https://dataview.ncbi.nlm.nih.gov/object/PRJNA1320650?reviewer=tbcruedh9ophhbofujpjm4g0ta (accessed on 16 March 2026)) and are publicly available as of the date of publication. Original Western blot and microscopy data reported in this paper will be shared by the lead contact upon request. Any additional information required to reanalyze the data reported in this paper is available from the corresponding author upon request.

## Supplementary Materials

The following supporting information can be downloaded at: https://www.mdpi.com/article/10.3390/antiox15040438/s1. Figure S1. ACSL4 expression increases with the progression of ALD, related to Figure 1. (A) KEGG enrichment analysis of DEGs in alcoholic liver disease from the GSE28619 dataset. (B) Cell viability and corresponding IC_50_ values of HepG2, AML-12, and PMHs treated with EtOH (25, 50, 100, 200, 400, 800 mM) for 24 h. (C) Quantification of proteins in Figure 1G. (D) Quantification of proteins in Figure 1I. Data are represented as the mean ± SD, * *p* < 0.05, ** *p* < 0.01, *** *p* < 0.001 by unpaired two-tailed Student’s *t*-test (C,D) and one-way ANOVA test (B). Figure S2. The construction and verification of *Acsl4*^flox^ and *Acsl4*^HKO^ mice and stable ACSL4-knockdown cell lines, related to Figure 2. (A) Schematic diagram of the strategy for generating and breeding *Acsl4*^flox^ and *Acsl4*^HKO^ mice using CRISPR-Cas9 technology. (B,C) Relative mRNA (*n* = 10, (B)) and protein levels (C) of ACSL4 in PMHs isolated from livers of the Gao-Binge model *Acsl4*^flox^ and *Acsl4*^HKO^ mice. (D) Representative IHC staining (left) and quantification (right) of ACSL4 in livers from Gao-Binge model *Acsl4*^flox^ and *Acsl4*^HKO^ mice. Scale bar, 50 μm (*n* = 6). (E) Relative mRNA levels of ACSL4 in HepG2 cells following transduction with shNC, sh*ACSL4*-1, sh*ACSL4*-2. (F) Protein levels (left) and quantification (right) of ACSL4 in HepG2 cells following transduction with shNC, sh*ACSL4*-1, and sh*ACSL4*-2. Data are represented as the mean ± SD, * *p* <0.05, ** *p* < 0.01, *** *p* < 0.001 by unpaired two-tailed Student’s *t*-test (B–D) and one-way ANOVA test (E,F). Figure S3. Hepatocyte-specific *Acsl4* abrogation ameliorates pathological features of alcoholic liver disease, related to Figure 2. A–F. Gao-Binge model *Acsl4*^flox^ and *Acsl4*^HKO^ mice. (A) Body weight. (B) Liver/body weight ratio. (C) Proportional analysis of hepatic myeloid cell populations. Frequencies of myeloid cells (CD45^+^CD11b^+^), mature dendritic cells (CD11b^+^MHCII^hi^), M1 macrophages (CD11b^+^F4/80^hi^CD86^+^), M2 macrophages (CD11b^+^F4/80^hi^CD206^+^) among CD11b^+^ cells. All populations were gated on live, singlet, CD45^+^ immune cells. (D) Hepatic protein levels of pro-inflammatory cytokines (TNF-α, IL-6, IL-1β, MCP-1) measured by ELISA (*n* = 8). (E) Relative mRNA levels of genes related to alcohol metabolism (*Adh1*, *Aldh2*, and *Cyp2e1*) in livers (*n* = 10). (F) GSEA enrichment plots (alcohol dehydrogenase (NADP+) activity, aldehyde dehydrogenase (NAD+) activity) in our liver RNA-seq. Data are presented as the mean ± SD, * *p* < 0.05, ** *p* < 0.01, *** *p* < 0.001 by unpaired two-tailed Student’s *t*-test (E), one-way ANOVA test (B–D) and two-way ANOVA test (A). Figure S4. ACSL4 regulates lipid metabolism in alcoholic liver disease, related to Figure 3. (A) GSEA enrichment plots (fatty acid oxidation, fatty acyl-CoA binding, long-chain acid transporter activity, positive regulation of fatty acid biosynthetic process, and fatty acid transport) in our liver RNA-seq. (B) Quantification of proteins in Figure 3C. (C) Quantification of non-mitochondrial oxygen consumption and basal respiration of HepG2 cells treated with EtOH (100 mM, 24 h) following transduction with shNC or sh*ACSL4*. Data are presented as the mean ± SD, * *p* < 0.05, ** *p* < 0.01, *** *p* < 0.001 by unpaired two-tailed Student’s *t*-test (B,C). Figure S5. ACSL4 regulates redox homeostasis in ALD via γ-GC, related to Figure 4. (A) GSEA enrichment plots (glutamine metabolic process, glutathione peroxidase activity) in our liver RNA-seq from Gao-Binge model *Acsl4*^flox^ and *Acsl4*^HKO^ mice. (B) Representative bright-field images of AML12 treated with EtOH (400 mM, 24 h) following transduction with shNC or sh*Acsl4*. Scale bar, 200 μm (*n* = 6). (C) Cell viability of HepG2 cells treated with EtOH (100–800 mM, 24 h) following transduction with plasmids pcDNA3.1-NC and pcDNA3.1-*ACSL4*. (D) LDH release assay of HepG2 cells treated with EtOH (24 h) following transduction with shNC or sh*ACSL4*. (E) MFI of DCFH-DA fluorescence in HepG2 cells treated with EtOH (400 mM, 24 h) and γ-GC (500 μM, 24 h). (F,G) Fluorescence intensity (F) and MFI (G) of BODIPY 581/591 C11 (FITC, oxidation state; PE/Texas Red, reduction state) in AML12 and HepG2 cells treated with EtOH (400 mM/100 mM, 24 h) and γ-GC (500 μM, 24 h). (H,I) MFI (H) and fluorescence intensity (I) of MitoSOX Red in HepG2 and AML12 cells treated with EtOH (400 mM/100 mM, 24 h) and γ-GC (500 μM, 24 h). (J) MFI of DCFH-DA fluorescence in HepG2 cells treated with EtOH (400 mM, 24 h) and BSO (1 mM, 24 h) following transduction with shNC or sh*ACSL4*. (K,L) Fluorescence intensity (K) and MFI (L) of BODIPY 581/591 C11 (FITC, oxidation state; PE/Texas Red, reduction state) in AML12 and HepG2 cells treated with EtOH (400 mM/100 mM, 24 h) and BSO (1 mM, 24 h) following transduction with shNC or sh*ACSL4*. (M,N) MFI (M) and fluorescence intensity (N) of MitoSOX Red in HepG2 and AML12 cells treated with EtOH (400 mM/100 mM, 24 h) and BSO (1 mM, 24 h) following transduction with shNC or sh*ACSL4*. (O) Cell viability of HepG2 cells treated with erastin (20 μM, 24 h) and BSO (1 mM, 24 h) following transduction with shNC or sh*ACSL4*. Data are presented as the mean ± SD, * *p* < 0.05, ** *p* < 0.01, *** *p* < 0.001 by unpaired two-tailed Student’s *t*-test (C,D), one-way ANOVA test (E–O). Figure S6. ACSL4 knockdown suppresses PTP4A1 and its mediated MAPK/NF-κB pathway by releasing γ-GC, related to Figure 5. (A) Docking energies of γ-GC with proteins encoded by the DEGs identified in our liver RNA-seq (ranked by binding energy, cutoff < −5.0 kcal/mol). (B) Quantification of proteins in Figure 5D (upper). Relative mRNA levels of PTP4A1 in AML12 cells treated with γ-GC (250, 500, 1000 μM), HDA (2.5, 5, 10 μM), m-CA (50, 100, 200 μM), DL-BSA (50, 100, 200 μM) for 24 h (bottom). (C) Docking energies of metabolites with PTP4A1. (D) Co-immunoprecipitation of ectopically expressed flag-ACSL4 and myc-PTP4A1 in HepG2 cells. (E) Quantification of proteins in Figure 5J. (F) Relative mRNA levels of *PTP4A1* in HepG2 cells following transduction with shNC, sh*ACSL4*-1, sh*ACSL4*-2. (G) Quantification of proteins in Figure 5L. (H) GSEA enrichment plots for MAPK pathways (JNK and ERK1/2) in our liver RNA-seq from Gao-Binge model *Acsl4*^flox^ and *Acsl4*^HKO^ mice. (I) Quantification of proteins in Figure 5N–P. Data are presented as the mean ± SD, * *p* < 0.05, ** *p* < 0.01, *** *p* < 0.001 by one-way ANOVA test (B,E–G,I). Figure S7. Efficacy and safety indicators of JMS-053, related to Figure 6. B-G. JMS-053-treated Gao-Binge model mice. (A) Quantification of proteins in Figure 6C. (B) Serum ALT and AST levels on day 10. (C) Body weight. (D) Liver/body weight ratio. (E) Blood biochemistry parameters. (F) CBC parameters. (G) Quantification of proteins in Figure 6I. Data are presented as the mean ± SD, * *p* < 0.05, ** *p* < 0.01, *** *p* < 0.001 by unpaired two-tailed Student’s *t*-test (A,B), one-way ANOVA test (D–G) and two-way ANOVA test (C). Figure S8. Safety evaluation of JMS-053 based on histopathology, related to Figure 6. (A) Representative H&E staining of the heart, spleen, lung, and kidney. Scale bar, 100 μm (*n* = 6). (B) Histopathological assessment of major organs. Figure S9. Affinity measurement of screened compounds for ACSL4, related to Figure 7. (A) Docking energy of compounds with ACSL4. (B) Cell viability and corresponding EC_50_ values of AML12 and HepG2 cells treated with AA (0.2 mM, 24 h) or EtOH (400 mM, 24 h) and Dron. (C) MST fit results of Dron with ACSL4. Figure S10. Pharmacological screening of compounds targeting ACSL4, related to Figure 7. (A,B) Cell viability of AML12 and HepG2 cells treated with AA (0.2 mM, 24 h) and screened compounds. Data are presented as the mean ± SD, * *p* < 0.05, ** *p* < 0.01, *** *p* < 0.001 by one-way ANOVA test (A,B). Figure S11. Efficacy and safety indicators of Dron, related to Figure 7. (A–C). Dron-treated Gao-Binge model mice. (A) Body weight. (B) Blood biochemistry parameters. (C) CBC parameters. Data are presented as the mean ± SD, * *p* < 0.05, ** *p* < 0.01, *** *p* < 0.001 by one-way ANOVA test (B,C) and two-way ANOVA test (A). Figure S12. Safety evaluation of Dron based on histopathology, related to Figure 7. (A) Representative H&E staining of the heart, spleen, lung, and kidney. Scale bar, 100 μm (*n* = 6). (B) Histopathological assessment of major organs. Table S1. The primer sequences for the targeted genes, related to all figures. Table S2. The primer sequences for gene identification, related to Figure S2. Table S3. The primer sequences for the shRNA lentivirus targeting ACSL4. Table S4. Key Source Table. Supplementary Materials and Methods.

## Abbreviations

The following abbreviations are used in this manuscript:

ALD	Alcoholic Liver Disease
ACSL4	Acyl-CoA Synthetase Long Chain Family Member 4
γ-GC	Gamma-Glutamylcysteine
PTP4A1	Protein Tyrosine Phosphatase Type IVA member 1
AH	Alcoholic Hepatitis
HCC	Hepatocellular Carcinoma
ROS	Reactive Oxygen Species
PUFA	Polyunsaturated Fatty Acids
LPO	Toxic Lipid Peroxide
AA	Arachidonic Acid
MASLD	Metabolic Dysfunction-associated Steatotic Liver Disease
ALT	Alanine Aminotransferase
AST	Aspartate Aminotransferase
TG	Triglycerides
TCHO	Total Cholesterol
CBC	Complete Blood Count
MDA	Malondialdehyde
SOD	Superoxide Dismutase
CAT	Catalase
GSH	Glutathione
H&E	Hematoxylin and Eosin
MPO	Myeloperoxidase
IHC	Immunohistochemistry
WSI	Whole-slide Scanned Images
CCK8	Cell Counting Kit-8
LDH	Lactate Dehydrogenase
CETSA	Cellular Thermal Shift Assay
MST	Microscale Thermophoresis
OCR	Oxygen Consumption Rate
GEO	Gene Expression Omnibus
GO	Gene Ontology
DEG	Differentially Expressed Gene
KEGG	Kyoto Encyclopedia of Genes and Genomes
TCGA	The Cancer Genome Atlas
PMH	Primary Mouse Hepatocyte
PA	Palmitic Acid
OA	Oleic Acid
GSEA	Gene Set Enrichment Analysis
BSO	Buthionine Sulfoximine
HDA	Hexadecanamide
m-CA	m-Coumaric Acid
DL-BSA	DL-Benzylsuccinic Acid
Co-IP	Co-Immunoprecipitation
PTP	Protein Tyrosine Phosphatase
TP	Total Protein
MAPK	Mitogen-Activated Protein Kinase
NF-κB	Nuclear Factor Kappa-B
