# Supplementary material for: Hepatic ACSL4 Loss Boosts Endogenous Gamma-Glutamylcysteine to Alleviate Alcoholic Liver Disease
Source: Antioxidants (Basel). 2026 Mar 31;15(4):438. doi: 10.3390/antiox15040438 (PMC13113857; doi:10.3390/antiox15040438)
Supplement: Supplementary file 1 [file antioxidants-15-00438-s001.zip › antioxidants-4168900-supplementary.pdf]

Supporting Information

For

Hepatic ACSL4 Loss Boosts Endogenous γ-Glutamylcysteine to Ameliorate Alcoholic Liver Disease

This file includes:

Supplementary Figures and Figure Legends S1 to S12

Supplementary Tables S1 to S4

Supplementary Materials and Methods

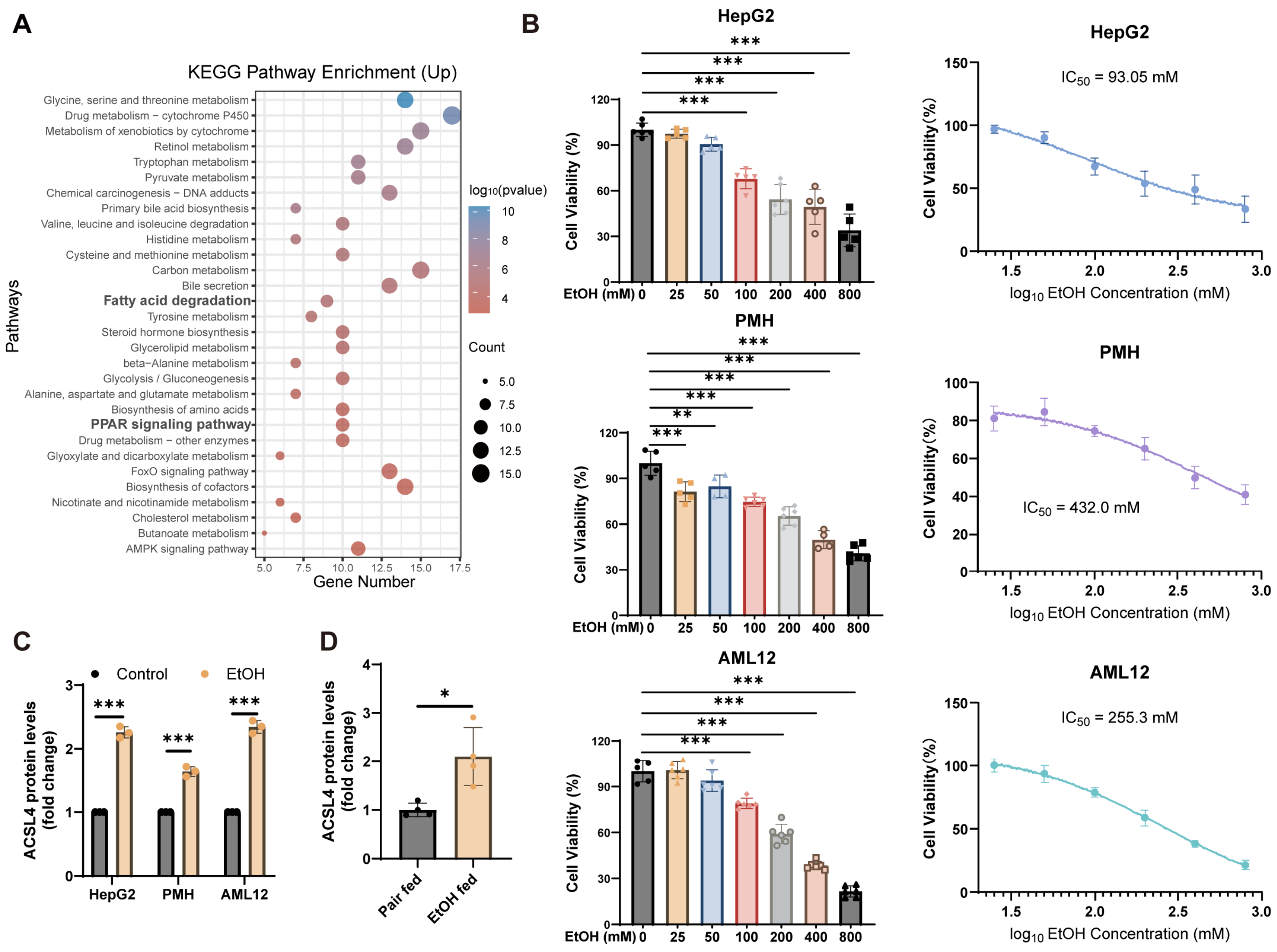

Figure S1. ACSL4 expression increases with the progression of ALD, related to figure 1. (A) KEGG enrichment analysis of DEGs in alcoholic liver disease from the GSE28619 dataset. (B) Cell viability and corresponding IC_{50} values of HepG2, AML-12, and PMHs treated with EtOH (25, 50, 100, 200, 400, 800 mM) for 24 h. (C) Quantification of proteins in figure 1G. (D) Quantification of proteins in figure 1I. Data are represented as the mean ± SD, *$p < 0.05$, **$p < 0.01$, ***$p < 0.001$ by unpaired two-tailed Student's *t*-test (C, D) and one-way ANOVA test (B).

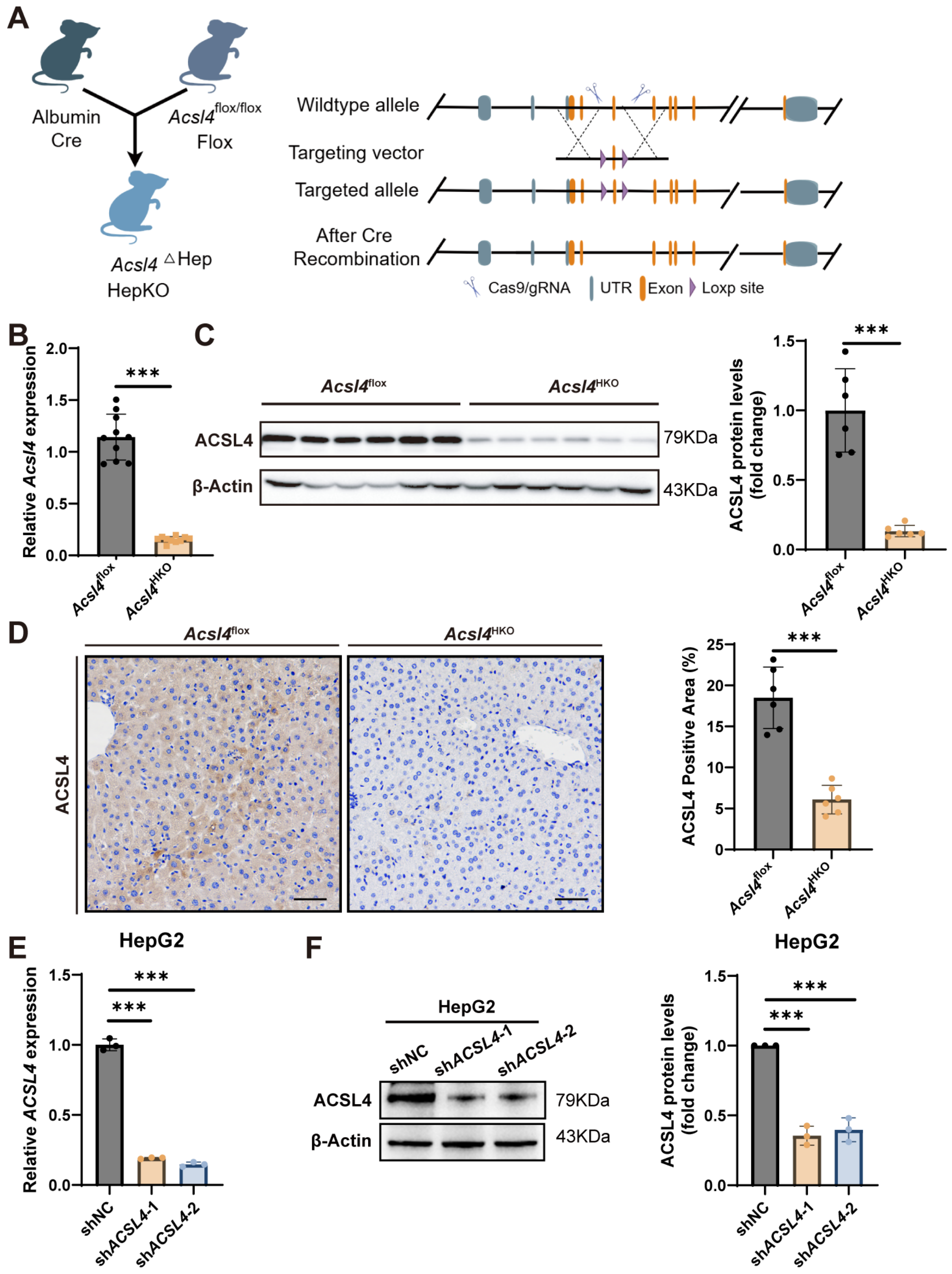

Figure S2. The construction and verification of *Acsl4*^{flox} and *Acsl4*^{HKO} mice and stable *ACSL4*-knockdown cell lines, related to figure 2. (A) Schematic diagram of the strategy for generating and breeding *Acsl4*^{flox} and *Acsl4*^{HKO} mice using CRISPR-Cas9 technology. (B-C) Relative mRNA (*n*=10, B) and protein levels (C) of ACSL4 in PMHs isolated from livers of the Gao-Binge model *Acsl4*^{flox} and *Acsl4*^{HKO} mice. (D) Representative IHC staining (left) and quantification (right) of ACSL4 in livers from Gao-Binge model *Acsl4*^{flox} and *Acsl4*^{HKO} mice. Scale bar, 50 μm (*n*=6). (E) Relative mRNA levels of *ACSL4* in HepG2 cells following transduction with shNC, sh*ACSL4*-1, sh*ACSL4*-2. (F) Protein levels (left) and quantification (right) of ACSL4 in HepG2 cells following transduction with shNC, sh*ACSL4*-1, and sh*ACSL4*-2. Data are represented as the mean ± SD, * p <0.05, **p < 0.01, ***p < 0.001 by unpaired two-tailed Student's *t*-test (B-D) and one-way ANOVA test (E, F).

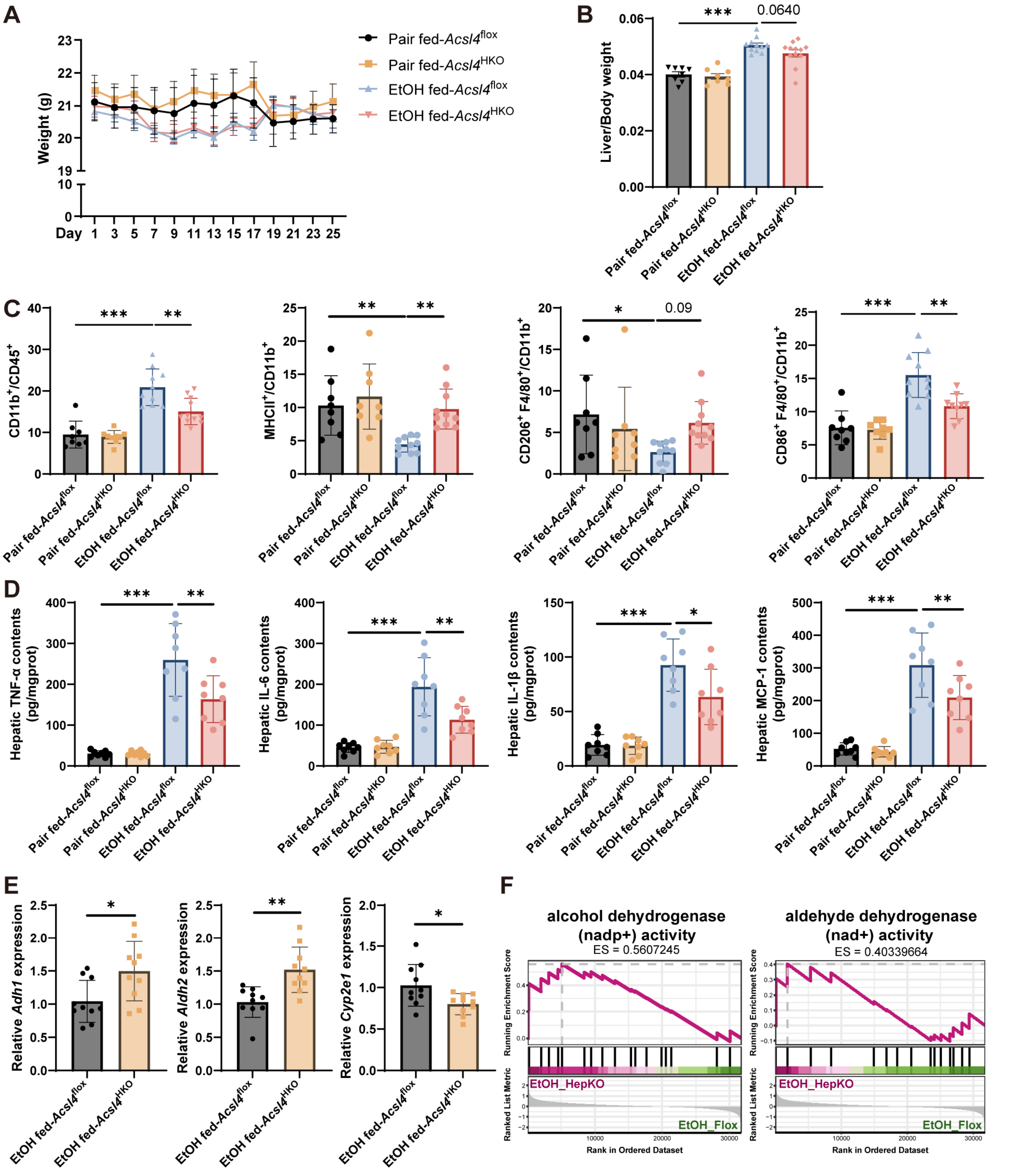

Figure S3. Hepatocyte-specific *Acsl4* abrogation ameliorates pathological features of alcoholic liver disease, related to figure 2. A-F. Gao-Binge model *Acsl4*^{flox} and *Acsl4*^{HKO} mice. (A) Body weight. (B) Liver/body weight ratio. (C) Proportional analysis of hepatic myeloid cell populations. Frequencies of myeloid cells (CD45$^+$CD11b$^+$), mature dendritic cells (CD11b$^+$MHCII^{hi}), M1 macrophages (CD11b$^+$F4/80^{hi}CD86$^+$), M2 macrophages (CD11b$^+$F4/80^{hi}CD206$^+$) among CD11b$^+$ cells. All populations were gated on live, singlet, CD45$^+$ immune cells. (D) Hepatic protein levels of pro-inflammatory cytokines (TNF-α, IL-6, IL-1β, MCP-1) measured by ELISA (n=8). (E) Relative mRNA levels of genes related to alcohol metabolism (*Adh1*, *Aldh2*, and *Cyp2e1*) in livers (n=10). (F) GSEA enrichment plots (alcohol dehydrogenase (NADP+) activity, aldehyde dehydrogenase (NAD+) activity) in our

liver RNA-seq. Data are presented as the mean ± SD, *$p < 0.05$, **$p < 0.01$, ***$p < 0.001$ by unpaired two-tailed Student's *t*-test (E), one-way ANOVA test (B-D) and two-way ANOVA test (A).

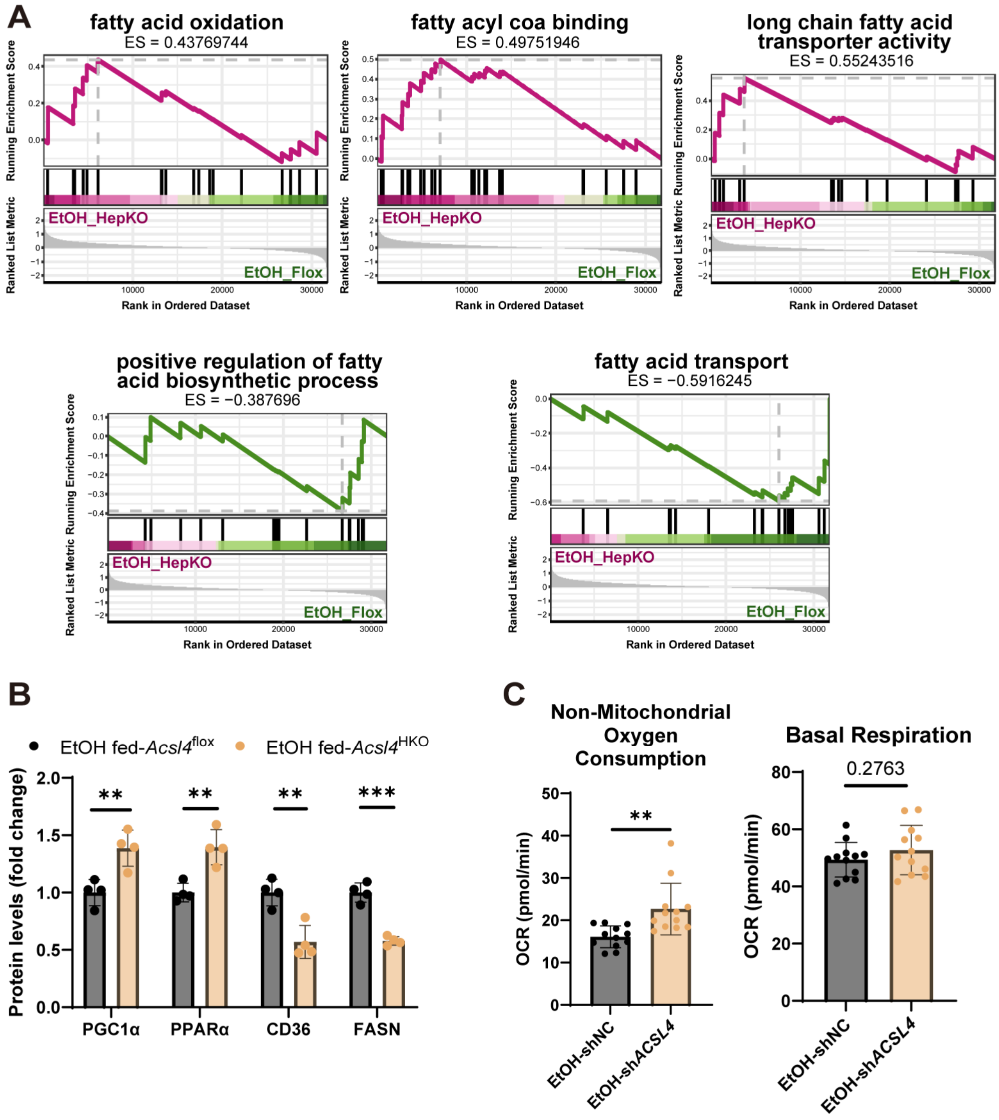

Figure S4. ACSL4 regulates lipid metabolism in alcoholic liver disease, related to figure 3. (A) GSEA enrichment plots (fatty acid oxidation, fatty acyl-CoA binding, long-chain acid transporter activity, positive regulation of fatty acid biosynthetic process, and fatty acid transport) in our liver RNA-seq. (B) Quantification of proteins in figure 3C. (C) Quantification of non-mitochondrial oxygen consumption and basal respiration of HepG2 cells treated with EtOH (100 mM, 24 h) following transduction with shNC or sh*ACSL4*. Data are presented as the mean ± SD, *$p < 0.05$, **$p < 0.01$, ***$p < 0.001$ by unpaired two-tailed Student's *t*-test (B, C).

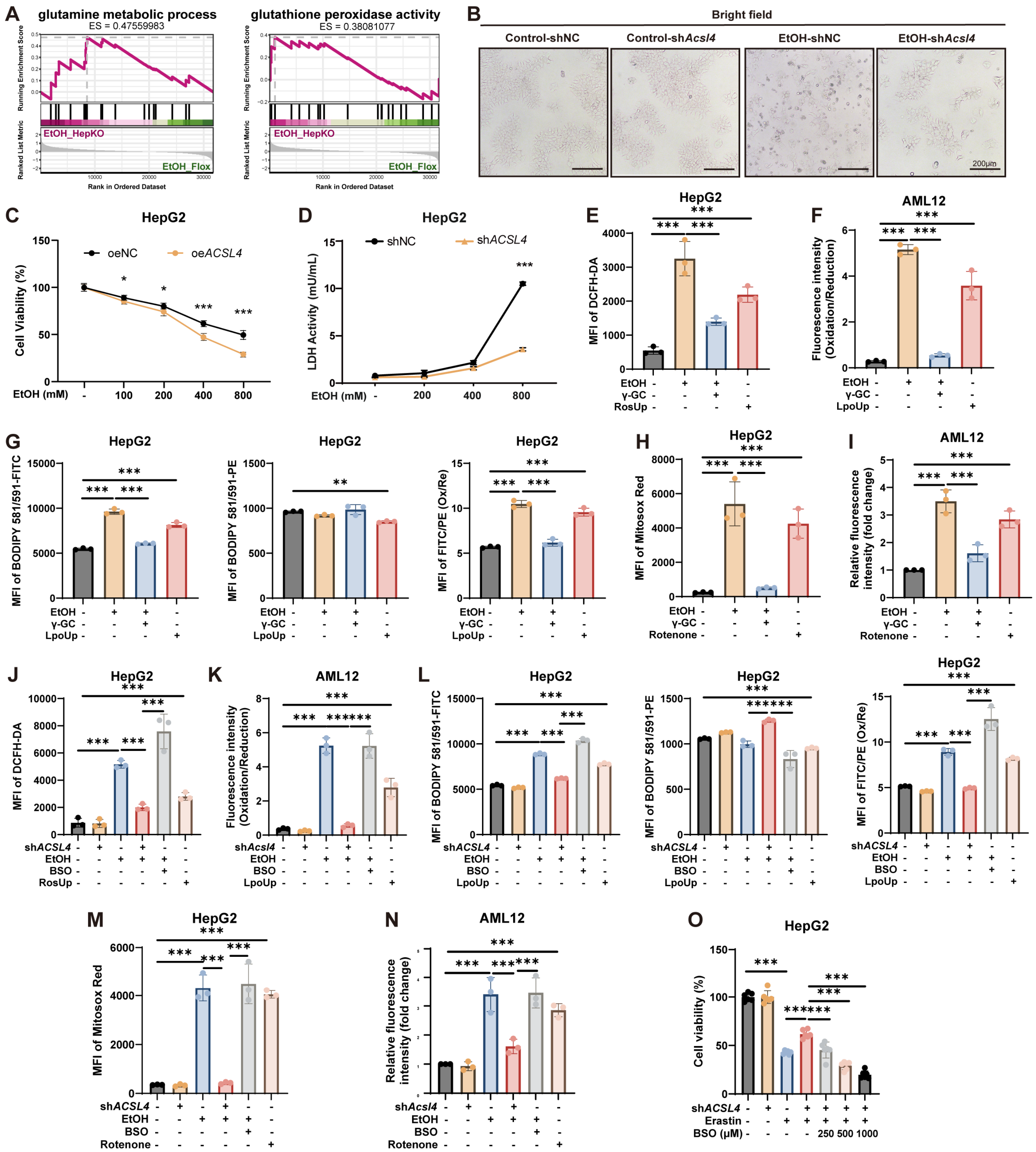

Figure S5. ACSL4 regulates redox homeostasis in ALD via γ-GC, related to figure 4. (A) GSEA enrichment plots (glutamine metabolic process, glutathione peroxidase activity) in our liver RNA-seq from Gao-Binge model *Acsl4*^{flox} and *Acsl4*^{HKO} mice. (B) Representative bright-field images of AML12 treated with EtOH (400 mM, 24 h) following transduction with shNC or sh*Acsl4*. Scale bar, 200 μm (*n*=6). (C) Cell viability of HepG2 cells treated with EtOH (100–800 mM, 24 h) following transduction with plasmids pcDNA3.1-NC and pcDNA3.1-*ACSL4*. (D) LDH release assay of HepG2 cells treated with EtOH (24 h) following transduction with shNC or sh*ACSL4*. (E) MFI of DCFH-DA fluorescence in HepG2 cells treated with EtOH (400 mM, 24 h) and γ-GC (500 μM, 24 h). (F-G) Fluorescence intensity (F) and MFI (G) of BODIPY 581/591 C11 (FITC, oxidation state; PE/Texas Red,

reduction state) in AML12 and HepG2 cells treated with EtOH (400 mM/100 mM, 24 h) and γ-GC (500 μM, 24 h). (H-I) MFI (H) and fluorescence intensity (I) of MitoSOX Red in HepG2 and AML12 cells treated with EtOH (400 mM/100 mM, 24 h) and γ-GC (500 μM, 24 h). (J) MFI of DCFH-DA fluorescence in HepG2 cells treated with EtOH (400 mM, 24 h) and BSO (1 mM, 24 h) following transduction with shNC or sh*ACSL4*. (K-L) Fluorescence intensity (K) and MFI (L) of BODIPY 581/591 C11 (FITC, oxidation state; PE/Texas Red, reduction state) in AML12 and HepG2 cells treated with EtOH (400 mM/100 mM, 24 h) and BSO (1 mM, 24 h) following transduction with shNC or sh*ACSL4*. (M-N) MFI (M) and fluorescence intensity (N) of MitoSOX Red in HepG2 and AML12 cells treated with EtOH (400 mM/100 mM, 24 h) and BSO (1 mM, 24 h) following transduction with shNC or sh*ACSL4*. (O) Cell viability of HepG2 cells treated with erastin (20 μM, 24 h) and BSO (1 mM, 24 h) following transduction with shNC or sh*ACSL4*. Data are presented as the mean ± SD, *$p < 0.05$, **$p < 0.01$, ***$p < 0.001$ by unpaired two-tailed Student's *t*-test (C, D), one-way ANOVA test (E-O).

A

Protein	Small molecule	Docking binding energy
NMDE4	Gamma_L_glutamyl_L_cysteine	-5.7 kcal/mol
PLOD2	Gamma_L_glutamyl_L_cysteine	-5.6 kcal/mol
SC5A1	Gamma_L_glutamyl_L_cysteine	-5.6 kcal/mol
VIL1	Gamma_L_glutamyl_L_cysteine	-5.6 kcal/mol
TP4A1	Gamma_L_glutamyl_L_cysteine	-5.5 kcal/mol
MDR1	Gamma_L_glutamyl_L_cysteine	-5.4 kcal/mol
APJ	Gamma_L_glutamyl_L_cysteine	-5.4 kcal/mol
B3GT5	Gamma_L_glutamyl_L_cysteine	-5.4 kcal/mol
CLD7	Gamma_L_glutamyl_L_cysteine	-5.4 kcal/mol
DDX3Y	Gamma_L_glutamyl_L_cysteine	-5.4 kcal/mol
FAM83E	Gamma_L_glutamyl_L_cysteine	-5.4 kcal/mol
HTRA1	Gamma_L_glutamyl_L_cysteine	-5.4 kcal/mol
SEM7A	Gamma_L_glutamyl_L_cysteine	-5.4 kcal/mol
CAH1	Gamma_L_glutamyl_L_cysteine	-5.3 kcal/mol
MUC6	Gamma_L_glutamyl_L_cysteine	-5.3 kcal/mol
BIRC1	Gamma_L_glutamyl_L_cysteine	-5.2 kcal/mol
PLS1	Gamma_L_glutamyl_L_cysteine	-5.2 kcal/mol
PTPRZ	Gamma_L_glutamyl_L_cysteine	-5.2 kcal/mol
SCD	Gamma_L_glutamyl_L_cysteine	-5.2 kcal/mol
AGRG7	Gamma_L_glutamyl_L_cysteine	-5.1 kcal/mol
KIF1A	Gamma_L_glutamyl_L_cysteine	-5.1 kcal/mol
NOS1	Gamma_L_glutamyl_L_cysteine	-5.1 kcal/mol
CTL4	Gamma_L_glutamyl_L_cysteine	-5.1 kcal/mol
SPIT1	Gamma_L_glutamyl_L_cysteine	-5.1 kcal/mol
STAR	Gamma_L_glutamyl_L_cysteine	-5.1 kcal/mol
CP1B1	Gamma_L_glutamyl_L_cysteine	-5.0 kcal/mol
EPCAM	Gamma_L_glutamyl_L_cysteine	-5.0 kcal/mol
FRMD5	Gamma_L_glutamyl_L_cysteine	-5.0 kcal/mol
FUT5	Gamma_L_glutamyl_L_cysteine	-5.0 kcal/mol
PDLI4	Gamma_L_glutamyl_L_cysteine	-5.0 kcal/mol
SMCA1	Gamma_L_glutamyl_L_cysteine	-5.0 kcal/mol
PDLI4	Gamma_L_glutamyl_L_cysteine	-5.0 kcal/mol

B

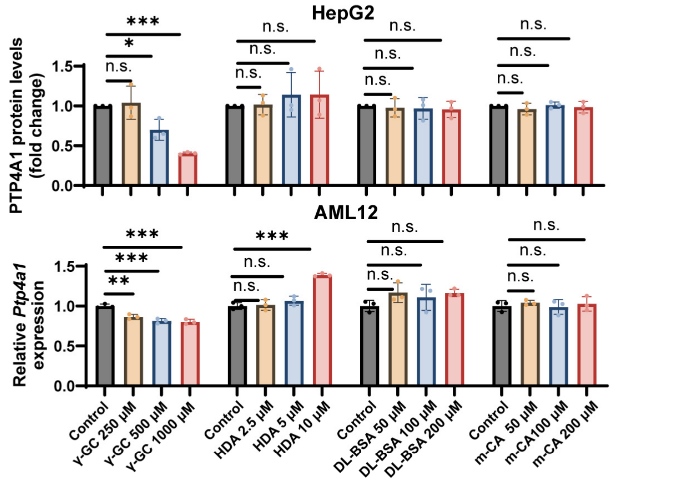

C

Protein	Small molecule	Docking binding energy
TP4A1	Gamma_L_glutamyl_L_cysteine	-5.5 kcal/mol
TP4A1	DL_Benzylsuccinic acid	-6.2 kcal/mol
TP4A1	M_Coumaric Acid	-5.1 kcal/mol
TP4A1	Hexadecanamide	-3.5 kcal/mol

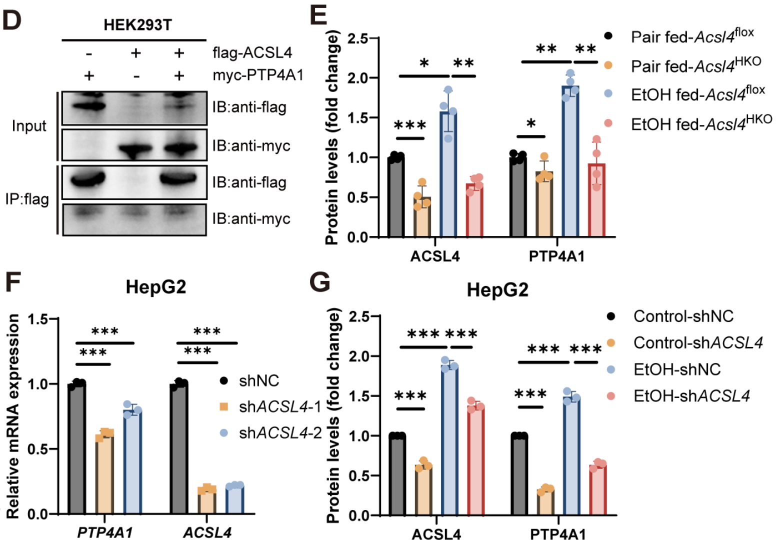

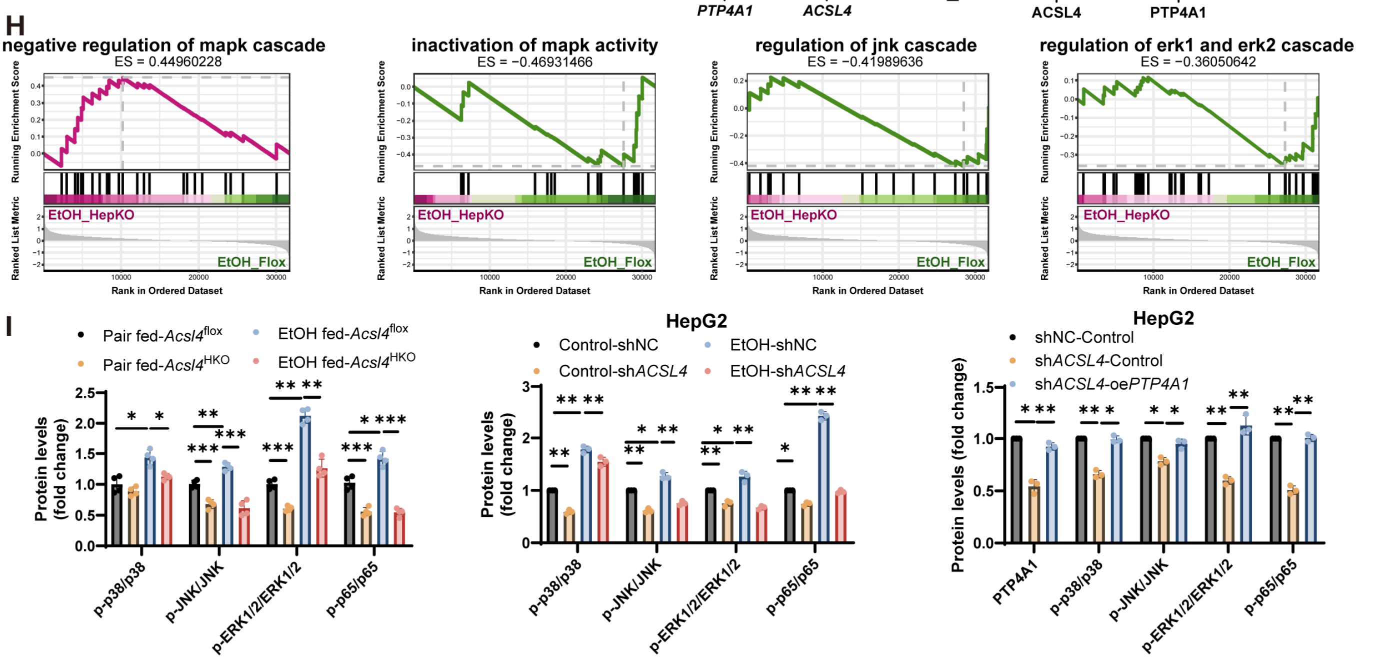

Figure S6. *ACSL4* knockdown suppresses PTP4A1 and its mediated MAPK/NF-κB pathway by releasing γ-GC, related to figure 5. (A) Docking energies of γ-GC with proteins encoded by the DEGs identified in our liver RNA-seq (ranked by binding

energy, cutoff < -5.0 kcal/mol). (B) Quantification of proteins in figure 5D (upper). Relative mRNA levels of *PTP4A1* in AML12 cells treated with γ-GC (250, 500, 1000 μM), HDA (2.5, 5, 10 μM), m-CA (50, 100, 200 μM), DL-BSA (50, 100, 200 μM) for 24 h (bottom). (C) Docking energies of metabolites with PTP4A1. (D) Co-immunoprecipitation of ectopically expressed flag-ACSL4 and myc-PTP4A1 in HepG2 cells. (E) Quantification of proteins in figure 5J. (F) Relative mRNA levels of *PTP4A1* in HepG2 cells following transduction with shNC, sh*ACSL4*-1, sh*ACSL4*-2. (G) Quantification of proteins in figure 5L. (H) GSEA enrichment plots for MAPK pathways (JNK and ERK1/2) in our liver RNA-seq from Gao-Binge model $Acsl4^{flox}$ and $Acsl4^{HKO}$ mice. (I) Quantification of proteins in figure 5N-5P. Data are presented as the mean ± SD, $^{*}p < 0.05$, $^{**}p < 0.01$, $^{***}p < 0.001$ by one-way ANOVA test (B, E-G, I).

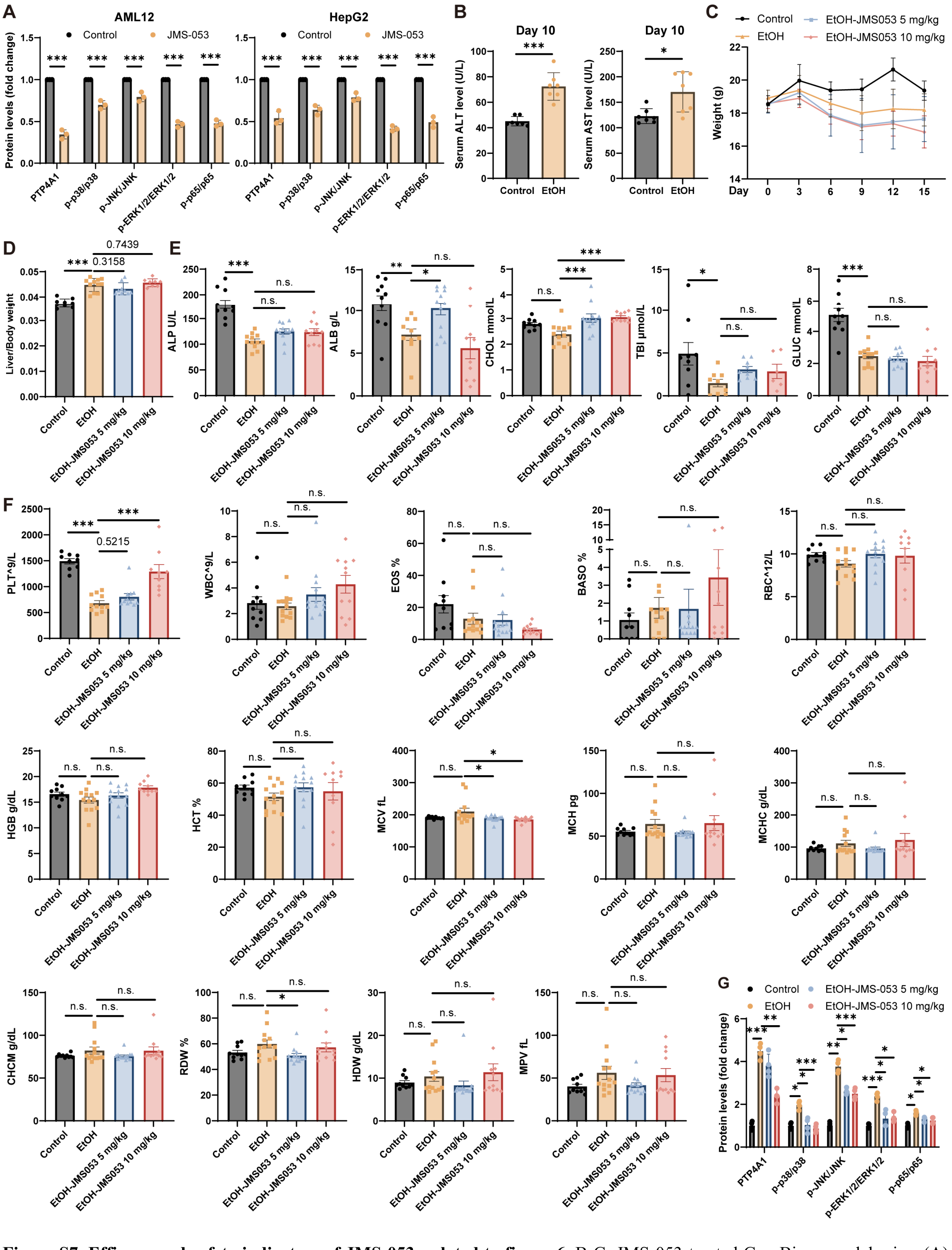

Figure S7. Efficacy and safety indicators of JMS-053, related to figure 6. B-G. JMS-053-treated Gao-Binge model mice. (A) Quantification of proteins in figure 6C. (B) Serum ALT and AST levels on day 10. (C) Body weight. (D) Liver/body weight ratio. (E) Blood biochemistry parameters. (F) CBC parameters. (G) Quantification of proteins in figure 6I. Data are presented as the mean

± SD, $*p < 0.05$, $**p < 0.01$, $***p < 0.001$ by unpaired two-tailed Student's *t*-test (A, B), one-way ANOVA test (D-G) and two-way ANOVA test (C).

A

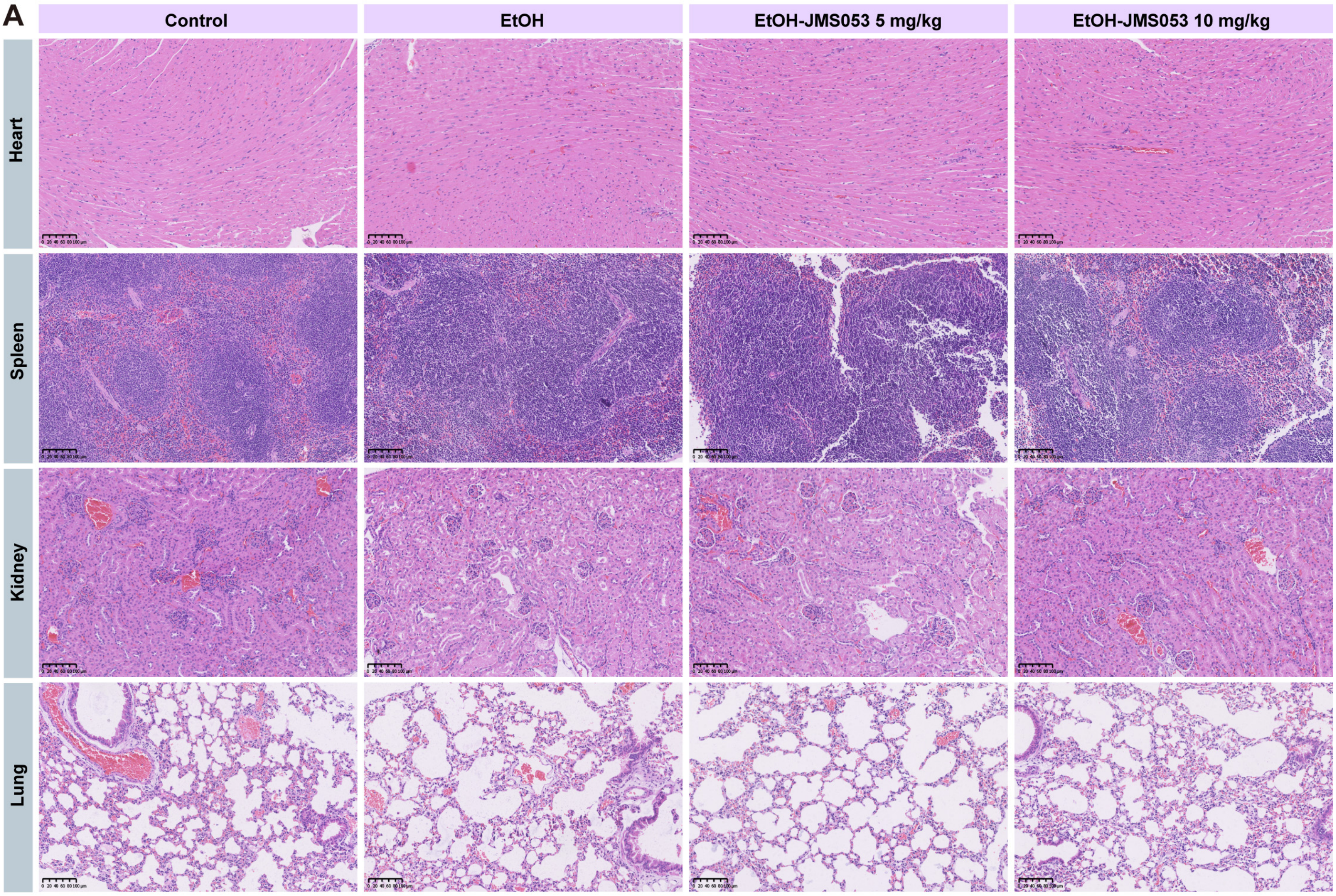

B

	Pathological findings	Control	EtOH	EtOH-Dron 50mg/kg	EtOH-Dron 100mg/kg
Heart	-	-	-	-	-
Spleen	-	-	-	-	-
Lung	-	-	-	-	-
Kidney	Dilation of the renal capsule (Bowman's space) with the presence of eosinophilic amorphous material, accompanied by shrinkage/retraction of the glomerular tuft (capillary loops). The renal tubules contain eosinophilic amorphous material.	-	+++	++	++

Figure S8. Safety evaluation of JMS-053 based on histopathology, related to figure 6. (A) Representative H&E staining of the heart, spleen, lung, and kidney. Scale bar, 100 μm (*n*=6). (B) Histopathological assessment of major organs.

A

Protein	Small molecule	Chemical Structure	Ligand protein docking binding energy
**ACSL4**	**Bosutinib**	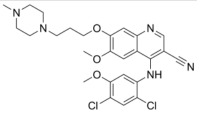	**-7.4 kcal/mol**
**ACSL4**	**Cabozantinib**	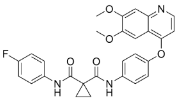	**-8.6 kcal/mol**
**ACSL4**	**Ceftriaxone**	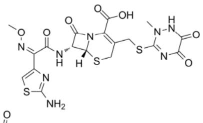	**-8.3 kcal/mol**
**ACSL4**	**Dronedarone**	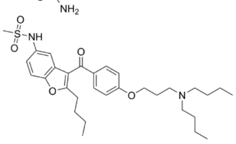	**-7.0 kcal/mol**
**ACSL4**	**Glimepiride**	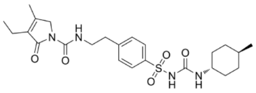	**-8.4 kcal/mol**
**ACSL4**	**Gliquidone**	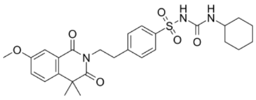	**-9.3 kcal/mol**
**ACSL4**	**Lutein**	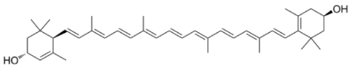	**-7.1 kcal/mol**
**ACSL4**	**Nintedanib**	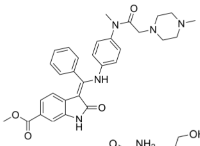	**-8.0 kcal/mol**
**ACSL4**	**Silodosin**	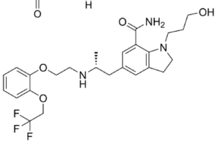	**-7.5 kcal/mol**
**ACSL4**	**Zafirlukast**	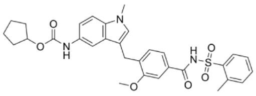	**-8.4 kcal/mol**

B

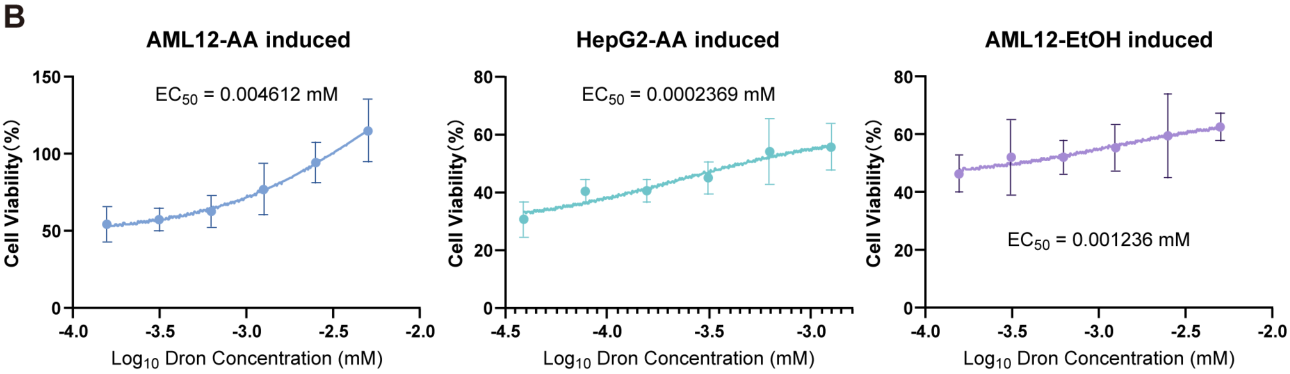

C

Fit Results (Kd)	
**Fit Model:**	Kd
**Bound**	942.42298
**Unbound**	870.7415004
**Kd**	1.1954E-05
**TargetConc**	5.63817E-05
**Standard Deviation:**	2.274081142
**Kd Confidence:**	±5.49257740565525E-06

Figure S9. Affinity measurement of screened compounds for ACSL4, related to figure 7. (A) Docking energy of compounds with ACSL4. (B) Cell viability and corresponding EC_{50} values of AML12 and HepG2 cells treated with AA (0.2 mM, 24 h) or EtOH (400 mM, 24 h) and Dron. (C) MST fit results of Dron with ACSL4.

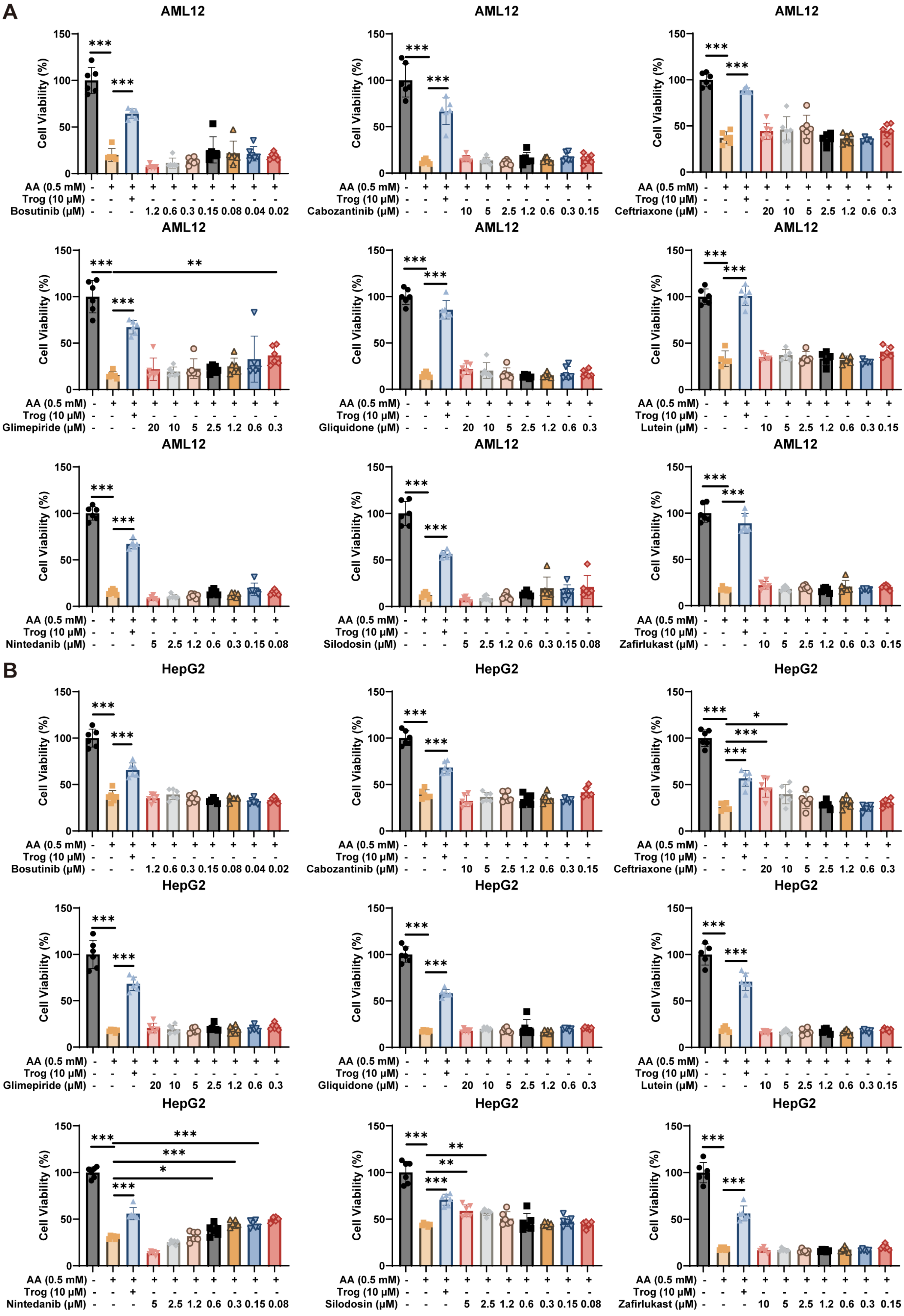
A
AML12
Cell Viability (%)
AA (0.5 mM)
Trog (10 μM)
Bosutinib (μM) - - - 1.2 0.6 0.3 0.15 0.08 0.04 0.02
Cabozantinib (μM) - - - 10 5 2.5 1.2 0.6 0.3 0.15
Ceftriaxone (μM) - - - 20 10 5 2.5 1.2 0.6 0.3
Glimepiride (μM) - - - 20 10 5 2.5 1.2 0.6 0.3
Gliquidone (μM) - - - 20 10 5 2.5 1.2 0.6 0.3
Lutein (μM) - - - 10 5 2.5 1.2 0.6 0.3 0.15
Nintedanib (μM) - - - 5 2.5 1.2 0.6 0.3 0.15 0.08
Silodosin (μM) - - - 5 2.5 1.2 0.6 0.3 0.15 0.08
Zafirlukast (μM) - - - 10 5 2.5 1.2 0.6 0.3 0.15
B
HepG2

Figure S10. Pharmacological screening of compounds targeting ACSL4, related to figure 7. (A-B) Cell viability of AML12 and HepG2 cells treated with AA (0.2 mM, 24 h) and screened compounds. Data are presented as the mean ± SD, *$p < 0.05$, **$p < 0.01$, ***$p < 0.001$ by one-way ANOVA test (A-B).

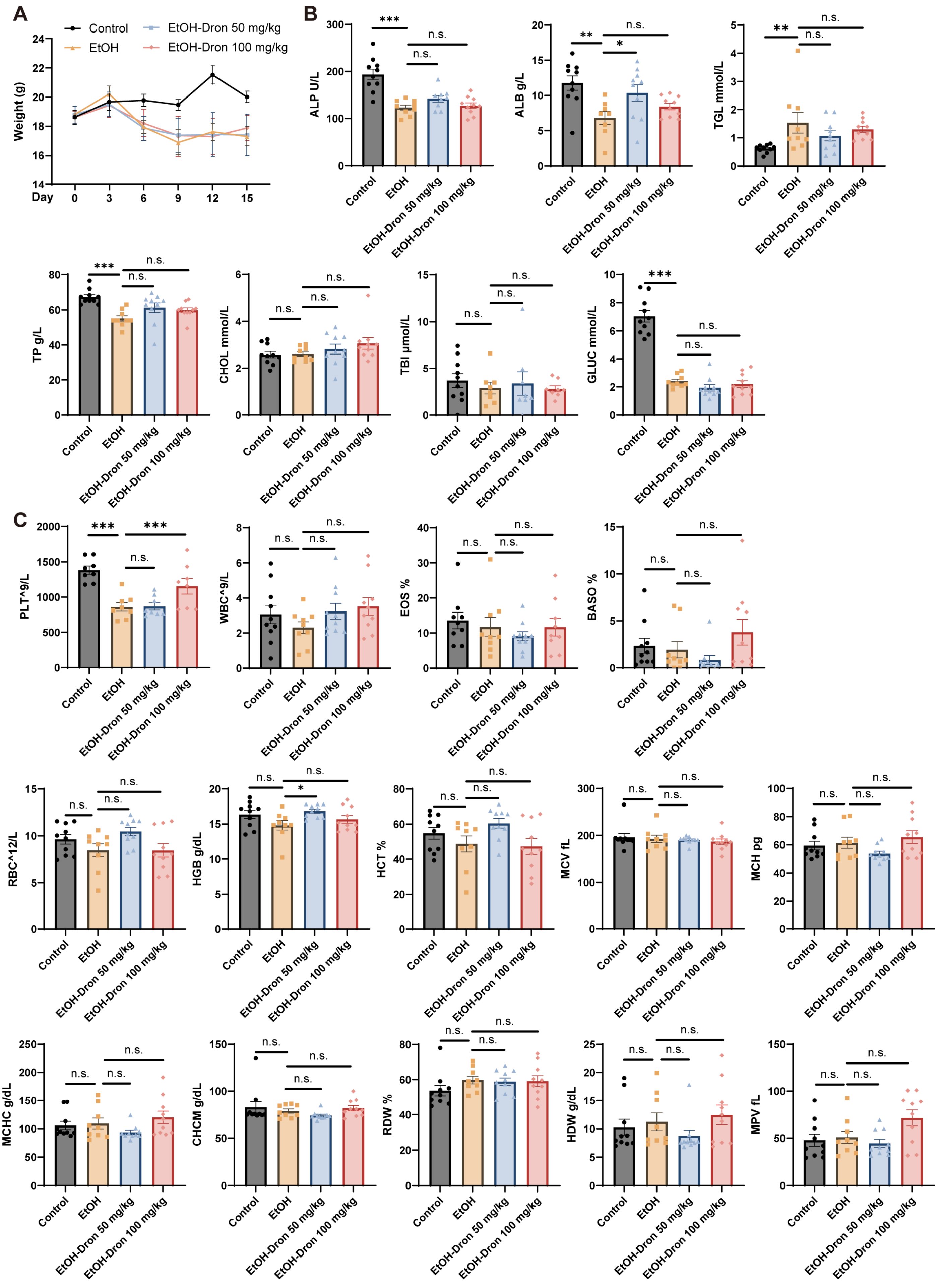
A
Control
EtOH
EtOH-Dron 50 mg/kg
EtOH-Dron 100 mg/kg
Weight (g)
Day
B
ALP U/L
ALB g/L
TGL mmol/L
TP g/L
CHOL mmol/L
TBI μmol/L
GLUC mmol/L
C
PLT^9/L
WBC^9/L
EOS %
BASO %
RBC^12/L
HGB g/dL
HCT %
MCV fL
MCH pg
MCHC g/dL
CHCM g/dL
RDW %
HDW g/dL
MPV fL
n.s.
*
**
***

Figure S11. Efficacy and safety indicators of Dron, related to figure 7. A-C. Dron-treated Gao-Binge model mice. (A) Body weight. (B) Blood biochemistry parameters. (C) CBC parameters. Data are presented as the mean ± SD, $*p < 0.05$, $**p < 0.01$, $***p < 0.001$ by one-way ANOVA test (B-C) and two-way ANOVA test (A).

A

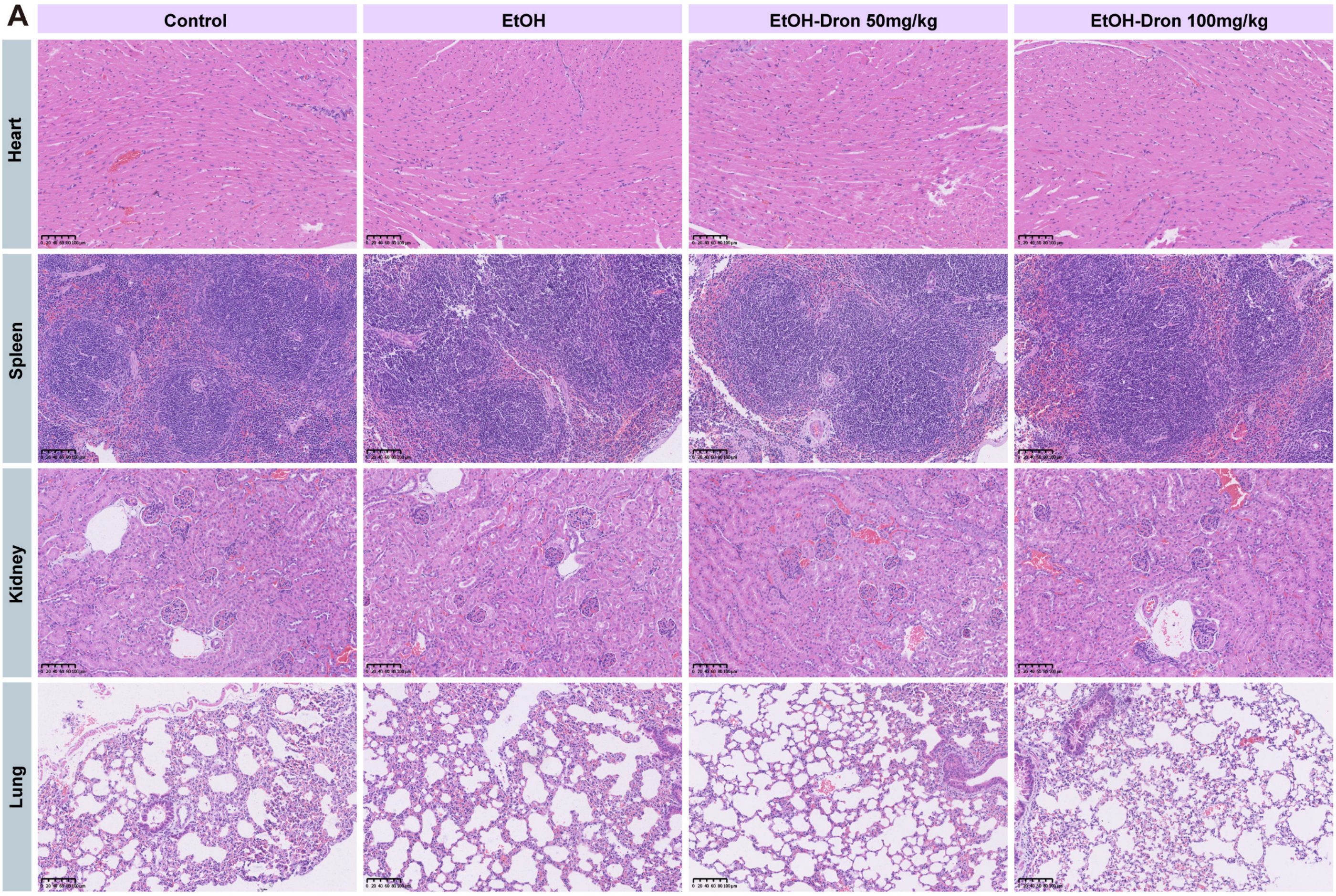

B

	Pathological findings	Control	EtOH	EtOH-Dron 50mg/kg	EtOH-Dron 100mg/kg
**Heart**	-	-	-	-	-
**Spleen**	-	-	-	-	-
**Lung**	-	-	-	-	-
**Kidney**	Dilation of the renal capsule (Bowman's space) with the presence of eosinophilic amorphous material, accompanied by shrinkage/retraction of the glomerular tuft (capillary loops). The renal tubules contain eosinophilic amorphous material.	-	+++	++	+

Figure S12. Safety evaluation of Dron based on histopathology, related to figure 7. (A) Representative H&E staining of the heart, spleen, lung, and kidney. Scale bar, 100 μm (*n*=6). (B) Histopathological assessment of major organs.

Table S1. The primer sequences for the targeted genes, related to all figures.

Refseq	Gene	Sequence 5'---3'
NM_001101.5	h*ACTIN*-F	GCGTGACATTAAGGAGAAG
	h*ACTIN*-R	GAAGGAAGGCTGGAAGAG
NM_001318509.1	h*ACSL4*-F	CATCCCTGGAGCAGATACTCT
	h*ACSL4*-R	TCACTTAGGATTTCCCTGGTCC
NM_001385254.1	h*PTP4A1*-F	GGCGTGGAGCTTTTAACAGC
	h*PTP4A1*-R	GCCGCATTTTAGGACGATACTT
NM_000576.2	h*IL-1β*-F	TTCGACACATGGGATAACGAGG
	h*IL-1β*-R	TTTTTGCTGTGAGTCCCGGAG
NM_007393.5	m*Actin*-F	GGCTGTATTCCCCTCCATCG
	m*Actin*-R	CCAGTTGGTAACAATGCCATGT
NM_001033600.1	m*Acsl4*-F	CTCACCATTATATTGCTGCCTGT
	m*Acsl4*-R	TCTCTTTGCCATAGCGTTTTTCT
NM_008361.4	m*Il-1β*-F	GAAATGCCACCTTTTGACAGTG
	m*Il-1β*-R	TGGATGCTCTCATCAGGACAG
NM_013693.3	m*Tnf*-α-F	GACGTGGAACTGGCAGAAGAG
	m*Tnf-α*-R	TTGGTGGTTTGTGAGTGTGAG
NM_001314054.1	m*Il-6*-F	CCAAGAGGTGAGTGCTTCCC
	m*Il-6*-R	CTGTTGTTCAGACTCTCTCCCT
NM_011333.3	m*Ccl2*-F	TTAAAAACCTGGATCGGAACCAA
	m*Ccl2*-R	GCATTAGCTTCAGATTTACGGGT
NM_008176.3	m*Cxcl1*-F	CTGGGATTCACCTCAAGAACATC
	m*Cxcl1*-R	CAGGGTCAAGGCAAGCCTC
NM_009140.2	m*Cxcl2*-F	CCAACCACCAGGCTACAGG
	m*Cxcl2*-R	GCGTCACACTCAAGCTCTG
NM_009141.3	m*Cxcl5*-F	TCCAGCTCGCCATTCATGC
	m*Cxcl5*-R	TTGCGGCTATGACTGAGGAAG
NM_013654.3	m*Ccl7*-F	GCTGCTTTCAGCATCCAAGTG
	m*Ccl7*-R	CCAGGGACACCGACTACTG
NM_007988.3	m*Fasn*-F	AAGGACCTGTCTAGGTTTGATGC

	m*Fasn*-R	TGGCTTCATAGGTGACTTCCA
NM_001313979.1	m*Srebp-1c*-F	CGGAACCATCTTGGCAACAGT
	m*Srebp-1c*-R	CGCTTCTCAATGGCGTTGT
NM_009127.4	m*Scd1*-F	TTCCTACCTGCAAGTTCTACACC
	m*Scd1*-R	CCGAGCTTTGTAAGAGCGGT
NM_011144.6	m*Pparα*-F	ATGGTGGACACGGAAAGCC
	m*Pparα*-R	CGATGGATTGCGAAATCTCTTGG
NM_013495.2	m*Cpt1α*-F	ATCAATCGGACTCTGGAAACGG
	m*Cpt1α*-R	TCAGGGAGTAGCGCATGGT
NM_133904.3	m*Acc2*-F	AGAAGACAAGAAGCAGGCAAAC
	m*Acc2*-R	GTAGACTCACGAGATGAGCCA
NM_011200.3	m*Ptp4a1*-F	CAATCCAACCAATGCGACCTT
	m*Ptp4a1*-R	GAGTAGTGTCGTAAGTTGCTTCA
NM_007409.3	m*Adh1*-F	GCAAAGCTGCGGTGCTATG
	m*Adh1*-R	TCACACAAGTCACCCCTTCTC
NM_001308450.1	m*Aldh2*-F	GAGGACTGTGTTGGGAGGTC
	m*Aldh2*-R	GTAGGTCCGGTCCCGTTC
NM_021282.3	m*Cyp2e1*-F	CGTTGCCTTGCTTGTCTGGA
	m*Cyp2e1*-R	AAGAAAGGAATTGGGAAAGGTCC

Table S2. The primer sequences for gene identification, related to figure S2.

Gene	Sequence 5'---3'
m*Alb*-Cre-F	TGGCAAACATACGCAAGGG
m*Alb*-Cre-R	CGGCAAACGGACAGAAGCA
m*Acsl4*-ko-F	AGTTAGCAGAGGGAGGCTGAATCT
m*Acsl4*-ko-R	ACCTCAGGATCTCAATCAAGTCAG

Table S3. The primer sequences for the shRNA lentivirus targeting ACSL4.

Primers	Sequence 5'---3'
LV2N-*ACSL4*-homo-1	GCAGTAGTTCATGGGCTAAAT
LV2N-*ACSL4*-homo-2	GCAGAGATATCTTGCTTTACC
LV2N-NC-homo	TTCTCCGAACGTGTCACGT
Mus *Acsl4*-shRNA-1	gcAGAAGATTATTGTGTTGAT

Mus *Acsl4*-shRNA-2	gcAAGGTTCAAGAGATGAATT
Mus NC shRNA	TTCTCCGAACGTGTCACGT

Table S4. Key Source Table

REAGENT or RESOURCE	SOURCE	IDENTIFIER
Experimental models: Organisms/strains		
Acsl4-floxed mice	Shanghai Model Organisms	N/A
Alb-Cre mice	Shanghai Model Organisms	N/A
C57BL6/j mice	Gempharmatech	N000013
Experimental models: Cell lines		
Mouse HepG2 human hepatoma cell	NCACC	SCSP-510<br>RRID:CVCL_0027
Alpha Mouse Liver 12	NCACC	SCSP-654<br>RRID:CVCL_0140
Antibodies		
Zombie NIR™ Fixable Viability Kit	Biolegend	Cat#423105
PE anti-mouse CD45 (Clone 30-F11)	Biolegend	Cat#103105, RRID:AB_312970
FITC anti-mouse/human CD11b (Clone M1/70)	Biolegend	Cat# 101205, RRID:AB_312788
Brilliant Violet 421™ anti-mouse F4/80 (Clone BM8)	Biolegend	Cat# 123137, RRID:AB_2563102
Brilliant Violet 711™ anti-mouse Ly-6C (Clone HK1.4)	Biolegend	Cat#128037, RRID:AB_2562630
PE/Cyanine7 anti-mouse Ly-6G(Clone 1A8)	Biolegend	Cat#127617, RRID:AB_1877262
APC anti-mouse CD11c (Clone N418)	Biolegend	Cat#117309, RRID:AB_313778
PerCP/Cyanine5.5 anti-mouse I-A/I-E (Clone M5/114.15.2)	Biolegend	Cat#107625, RRID:AB_2191072
Anti-FACL4 antibody [EPR8640]	Abcam	Cat#ab155282 RRID:AB_2714020
Beta Actin Monoclonal antibody	Proteintech	Cat#66009-1-Ig, RRID:AB_2687938
PPARA Polyclonal antibody	Proteintech	Cat#15540-1-AP
Phospho-NF-κB p65 (Ser536) (93H1) Rabbit mAb	CST	Cat#3033, RRID:AB_331284

NF-κB p65 (D14E12) XP® Rabbit mAb	CST	Cat#8242, RRID:AB_10859369
CD36 Polyclonal antibody	Proteintech	Cat#18836-1-AP, RRID: AB_2876995
FASN Rabbit Polyclonal antibody	Proteintech	Cat#10624-1-AP, RRID: AB_2296771
PGC1a Monoclonal antibody	Proteintech	Cat#66369-1-Ig, RRID: AB_2881932
PTP4A1/PRL1 Polyclonal antibody	Proteintech	Cat#11508-1-AP, RRID:AB_2237951
Phospho-p38 (Thr180/Tyr182) Rb pAb	Wanleibio	Cat#WLP1576, RRID:AB_2922420
p38 Rb pAb	Wanleibio	Cat#WL00764, RRID:AB_2922419
ERK1/2 Polyclonal antibody	Proteintech	Cat#51068-1-AP, RRID:AB_2250380
Phospho-ERK1/2 (Thr202/Tyr204) Polyclonal antibody	Proteintech	Cat#28733-1-AP, RRID:AB_2881202
JNK Polyclonal antibody	Proteintech	Cat#17572-1-AP, RRID: AB_2879123
Phospho-JNK (Tyr185) Recombinant antibody	Proteintech	Cat#80024-1-RR, RRID:AB_2882943
F4/80 Polyclonal antibody	Proteintech	Cat#28463-1-AP, RRID:AB_2881149
MPO Polyclonal antibody	Proteintech	Cat#22225-1-AP, RRID:AB_2879037
Phosphatase inhibitor (100x)	Fdbio	Cat#FD1002
JetPRIME Transfection reagent	Polyplus	Cat#114-15
RNAiso Plus	Takara	Cat#9019
ChamQ SYBR qPCR Master Mix (Low ROX Premixed)	Vazyme	Cat#Q331
HiScript III RT SuperMix for qPCR (+gDNA wiper)	Vazyme	Cat#R323
Oil Red O	Sigma	Cat#O9755
Bovine Serum Albumin V (Fatty acid free)	Solarbio	Cat#A8850
Collagenase	Sigma	Cat#C0130
EGTA	Sigma	Cat#E0396
Cell lysis buffer for Western and IP without inhibitors	Beyotime	Cat#P0013J
Protease inhibitors	Fdbio	Cat#FD1001
Phosphatase inhibitors	Fdbio	Cat#FD1002
Insulin	Thermo Fisher	Cat#12585-014
MitoSOX Red	MCE	Cat#HY-D1055
BODIPY™ 581/591 C11	Thermo Fisher	Cat#D3861

Rotenone	TargetMol	Cat#T2970
RSL3	MCE	Cat#HY-100218A
JMS-053	TargetMol	Cat#T60396
Dronedarone hydrochloride tablets	CPSC	Cat#H20193291
0.25% Trypsin-EDTA (1X)	NCM Biotech	Cat#No:C100C1
Penicillin-Streptomycin (100X)	NCM Biotech	Cat#No:C100C5
MEM-ALPHA	VivaCell	Cat#C3060-0500
DMEM (High Glucose)	VivaCell	Cat:C3110-0500
DMEM/F12	KeyGEN BioTECH	Cat#No:KGM12500-500
Certified Fetal Bovine Serum	VivaCell	Cat#No:C04001-500
Critical Commercial Assays		
Triglyceride assay kit	Nanjing Jiancheng	Cat#A110-1-1
Total cholesterol assay kit	Nanjing Jiancheng	Cat#A111-1-1
Cell Counting Kit-8 assay kit	Beyotime	Cat#C0043
Alanine aminotransferase Assay Kit	Nanjing Jiancheng	Cat#C009-2-1
Aspartate aminotransferase Assay Kit	Nanjing Jiancheng	Cat#C010-2-1
Mouse Tumor necrosis factor α, TNF-α ELISA KIT	Cusabio	Cat#CSB-E04741m
Mini Sample Mouse Interleukin 6,IL-6 ELISA Kit	Cusabio	Cat#CSB-E04639m-MS
Mini Sample Mouse Interleukin 1β,IL-1β ELISA Kit	Cusabio	Cat#CSB-E08054m-MS
Mouse monocyte chemotactic protein 1/monocyte chemotactic and activating factor,MCP-1/MCAF ELISA kit	Cusabio	Cat#CSB-E07430m
Reduced glutathione (GSH) assay kit	Nanjing Jiancheng	Cat#A006-2-1

Superoxide Dismutase (SOD) assay kit (WST-1 method)	Nanjing Jiancheng	Cat# A001-3-1
Catalase (CAT) assay kit (Visible light)	Nanjing Jiancheng	Cat# A007-1-1
LDH Cytotoxicity Assay Kit	Beyotime	Cat#C0016
BCA Protein Quantification Kit	Vazyme	Cat#E112-02
Pico ECL western-blotting substrate	Fdbio	Cat#FD8000
Malondialdehyde (MDA) assay kit (TBA method)	Nanjing Jiancheng	Cat#A003-1-2
ROS Assay Kit	Beyotime	Cat#S0033S
Deposited data		
Bulk RNA-seq data of liver from human	GEO datasets	GSE28619
Bulk RNA-seq data of liver from human	GEO datasets	GSE103580
Bulk RNA-seq data of liver from human	GEO datasets	GSE59259
Bulk RNA-seq data of liver from *Acsl4*^{flox} and *Acsl4*^{HKO} mice	SRA	PRJNA1320650
Oligonucleotides		
Please see Table S1 for oligonucleotide sequences for RT-qPCR primers	N/A	N/A
Please see Table S2 for oligonucleotide sequences for gene identification	N/A	N/A
Please see Table S3 for oligonucleotide sequences for the shRNA lentivirus targeting ACSL4	N/A	N/A
Software and algorithms		
FlowJo v.10.CL	Flowjo	https://www.flowjo.com/ RRID:SCR_008520
Prism 8	Graphpad	http://www.graphpad.com RRID:SCR_002798
ImageJ	NIH	https://imagej.nih.gov/ij/ RRID:SCR_003070

Adobe Photoshop	PS	https://www.adobe.com/products/photoshop.html<br>RRID:SCR_014199
Adobe Illustrator	AI	http://www.adobe.com/products/illustrator.html<br>RRID:SCR_010279
Other		
Lieber-DeCarli Alcohol Liquid Diet	Trophic	Cat#TP4030D
Lieber-DeCarli Control Liquid Diet	Trophic	Cat#TP4030C
Attune NxT flow cytometry	Thermo Fisher	Attune NxT
Q3 Realtime PCR machine	Thermo Fisher	A28131
Multi-function microplate reader	Perkin Elmer Enspire	2300
Inverted fluorescence microscope	Leica	DMI3000B

Supplemantary Materials and Methods

Mouse experiments

All mice were cohoused in the specific pathogen–free (SPF) facility of the Center for New Drug Safety Evaluation and Research, China Pharmaceutical University for 1 week prior to the start of experiments and maintained in an environment with a 12 h light/dark cycle, temperature between 20 and 26°C, humidity at 40% – 70%. Anesthesia was induced by intraperitoneal injection of tribromoethanol (Avertin) at a dose of 250 mg/kg. All animal experiments were in accordance with protocols approved by the Institutional Animal Care and Use Committee of the Center for New Drug Safety Evaluation and Research, China Pharmaceutical University.

Cell culture

HepG2 (SCSP-510, RRID:CVCL_0027) and AML-12 (SCSP-654, RRID:CVCL_0140) cells were purchased from the Cell Resource Center, Shanghai Institutes for Biological Sciences, Chinese Academy of Sciences (Shanghai, China). All cells were cultured at 37°C and 5% CO2. HepG2 cells were cultured in Minimum Essential Medium Alpha (MEM-Alpha, C3060-0500, VivaCell) supplemented with 10% FBS. AML-12 cells were cultured in DMEM-F12 (KGM12500-500, KeyGEN BioTECH) medium containing ITS-G (1%; 41400045, Thermo Fisher) and dexamethasone (40 ng/mL; ID0170, Solarbio).

Histopathological analysis

For IHC staining, liver sections from mice (4 μm) were incubated with the corresponding primary antibodies overnight at 4°C. The next day, the sections were incubated with horseradish peroxidase (HRP)-conjugated secondary antibodies for 30 minutes at room temperature, followed by DAB staining. The slides were then subjected to hematoxylin staining, 1% hydrochloric acid alcohol differentiation, ammonia solution counterstaining, and neutral balsam sealing. Subsequently, the section images were captured using a microscope (Nexcope). ImageJ (RRID:SCR_003070) was utilized to measure the integrated density and area of the entire section.

Histopathological diagnosis was conducted under the guidance of an experienced pathologist.

RNA isolation and real-time quantitative PCR

Total RNA was extracted from snap-frozen liver tissues or cells using RNAiso Plus (9109, Takara). RNA was reverse-transcribed into cDNA using HiScript III RT SuperMix for qPCR (+gDNA wiper) (R323, Vazyme). Subsequently, RT-qPCR was conducted using ChamQ SYBR qPCR Master Mix (Low ROX Premixed) (Q331, Vazyme). β-*Actin* was used for qPCR normalization, and the relative levels of mRNA expression were calculated using the comparative cycle method ($2^{-\Delta\Delta Ct}$). All experiments were performed in triplicate. The primers used for RT-qPCR are shown in Supplementary Table S1.

Western blot analysis

Cells or tissues were lysed in RIPA buffer (P0013J, Beyotime) supplemented with protease and phosphatase inhibitors

(FD1001, FD1002, Fdbio) at a 1:100 ratio. After centrifugation at 12,000 rpm for 10 min at 4°C, protein concentrations were determined using the BCA Protein Quantification Kit (E112-02, Vazyme). Samples were then heated at 95°C for 15 minutes. Subsequently, samples were separated by SDS-PAGE electrophoresis and transferred onto PVDF membranes (Amersham International). After blocking, membranes were incubated with primary antibodies in blocking solution overnight at 4°C. After several washes in TBST, HRP-conjugated secondary antibodies corresponding to the primary antibodies were incubated at room temperature for 2 hours. Bands were developed using the Pico ECL developing kit (FD8000, Fdbio). The primary antibodies used are shown in Supplementary Table S4.

ELISA

Hepatic levels of TNF-α, IL-6, IL-1β, and MCP-1 were measured using commercial ELISA kits (Cusabio). Frozen liver tissues were homogenized and centrifuged to obtain supernatants. After protein quantification, samples were assayed in accordance with the manufacturer's instructions. Cytokine concentrations were normalized to total protein and expressed as pg/mg protein.

Bulk RNA-sequencing analysis

Total RNA was isolated and purified using TRIzol reagent (Invitrogen, Carlsbad, CA, USA) following the manufacturer's procedure. The RNA amount and purity of each sample were quantified using NanoDrop ND-1000 (NanoDrop, Wilmington, DE, USA). The RNA integrity was assessed by Bioanalyzer 2100 (Agilent, CA, USA) with RIN number >7.0, and confirmed by electrophoresis with denaturing agarose gel. Poly (A) RNA was purified from 1 μg total RNA using Dynabeads Oligo (dT) 25-61005 (Thermo Fisher, CA, USA) using two rounds of purification. Then the poly(A) RNA was fragmented into small pieces using Magnesium RNA Fragmentation Module (NEB, cat.e6150, USA) at 94 ℃ 5 − 7 min. Then the cleaved RNA fragments were reverse-transcribed to create the cDNA by SuperScript™ II Reverse Transcriptase (Invitrogen, cat. 1896649, USA), which were next used to synthesise U-labeled second-stranded DNAs with E. coli DNA polymerase I (NEB, cat.m0209, USA), RNase H (NEB, cat.m0297, USA) and dUTP Solution (Thermo Fisher, cat.R0133, USA). After the heat-labile UDG enzyme (NEB, cat.m0280, USA) treatment of the U-labeled second-stranded DNAs, the ligated products are amplified with PCR. At last, we performed the 2×150 bp paired-end sequencing (PE150) on an illumina Novaseq™ 6000 (LC-Bio Technology CO., Ltd., Hangzhou, China) following the vendor's recommended protocol.

fastp software (https://github.com/OpenGene/fastp) was used to remove the reads that contained adaptor contamination, low quality bases and undetermined bases with default parameter and verified sequence quality. We used HISAT2 (https://ccb.jhu.edu/software/hisat2) to map reads to the reference genome of Mus musculus GRCm38, Ensembl/v101. After the final transcriptome was generated, StringTie was used to estimate the expression levels of all transcripts. StringTie was used to perform expression level for mRNAs by calculating FPKM (FPKM = [total_exon_fragments / mapped_reads(millions) × exon_length(kB)]). The differentially expressed mRNAs were selected with fold change > 2 or fold change < 0.5 and with

parametric F-test comparing nested linear models (p value < 0.05) by R package edgeR (https://bioconductor.org/packages/release/bioc/html/edgeR.html).

Metabolomics analysis

Widely targeted metabolomic profiling was conducted to characterize the hepatic metabolic landscape in *Acsl4*^{HKO} (*n*=5) and *Acsl4*^{flox} (*n*=5) mice. The analysis was carried out by MetWare Biotechnology Co., Ltd.

For metabolite extraction, frozen tissues were thawed on ice, and 50 mg of each sample was homogenized in 1000 µL of ice-cold extraction solution (70% methanol with 1 µg/mL 2-Chlorophenylalanine as internal standard). After thorough mixing, the homogenate was centrifuged at 12,000×g for 10 min at 4 °C. The resulting supernatant was collected for subsequent LC-MS/MS analysis.

Chromatographic separation was performed on a Shimadzu UHPLC system equipped with a Waters ACQUITY UPLC HSS T3 C18 column (1.8 µm, 2.1 × 100 mm) maintained at 40 °C. The mobile phase consisted of water and acetonitrile, each containing 0.04% acetic acid, using the following gradient: 95% water at 0 min, linearly decreased to 5% at 11.0 min, held until 12.0 min, returned to initial conditions at 12.1 min, and re-equilibrated until 14.0 min. The flow rate was 0.4 mL/min with an injection volume of 2 µL.

Mass spectrometric detection was carried out on a QTRAP® instrument equipped with an electrospray ionization source operated at 500 °C and voltages of ±5500/4500 V (positive/negative mode). Gas parameters were set as follows: ion source gas I, 55 psi; gas II, 60 psi; curtain gas, 25 psi. High collision-induced dissociation was applied, and multiple reaction monitoring transitions were optimized with specific declustering voltages and collision energies.

Data processing was performed using Analyst® Software 1.6.3. Multivariate analyses including PCA and OPLS-DA were employed to identify group differences, complemented by univariate analyses such as fold-change (FC) and t-tests. Model robustness was assessed through 10-fold cross-validation and 1000-cycle permutation tests. Multiple testing correction was applied using the false discovery rate (FDR). Differential metabolites were defined as those with $|FC| > 2$, variable importance in projection (VIP) ≥ 1, and a corrected p-value < 0.05 in two-sided statistical tests.
